# Infective endocarditis complicated by shock: a systematic review and meta-analysis

**DOI:** 10.1007/s10741-025-10556-5

**Published:** 2025-08-30

**Authors:** Roxana-Bianca Pîrîianu-Masgras, Alexandre Mebazaa, Gianluigi Savarese, Emanuel Stoica, Oliviana Geavlete, Andrew P. Ambrosy, Elena-Laura Antohi, Mehmet Birhan Yilmaz, Razvan-Ilie Radu, Marianna Adamo, Beth A. Davison, Jan Biegus, Gad Cotter, Javed Butler, Sean P. Collins, Ovidiu Chioncel

**Affiliations:** 1 University of Medicine and Pharmacy Carol Davila, 8 Eroii Sanitari Blvd, 050474 Bucharest, Romania; 2 Department of Anesthesia & Critical Care, Lariboisière Hospital, AP-HP, Paris, France; 3 Division of Cardiology, Department of Medicine, Karolinska Institutet, Stockholm, Sweden; 4 1, Department of Cardiology, Emergency Institute for Cardiovascular Diseases “C.C.Iliescu”, 258 Fundeni St, 022328 Bucharest, Romania; 5 Department of Cardiology, Kaiser Permanente San Francisco Medical Center, San Francisco, CA USA; 6 Department of Cardiology, Faculty of Medicine, Dokuz Eylül University, Izmir, Turkey; 7 Institute of Cardiology, ASST Spedali Civili, Brescia, Italy; 8 UMR-S 942 (MASCOT), Université Paris Cité, INSERM, Paris, France; 9 Momentum Research Inc, Durham, NC USA; 10 Heart Initiative, Durham, NC USA; 11 Department of Cardiology, Clinical Department of Intensive Cardiac Care, Institute of Heart Diseases, Faculty of Medicine, Wroclaw Medical University, Wroclaw, Poland; 12 Baylor Scott and White Research Institute, Dallas, TX USA; 13 University of Mississippi Medical Center, Jackson, MS USA; 14 Department of Emergency Medicine, Vanderbilt University Medical Center, Nashville, TN USA; 15 Université Paris Cité, Paris, France; 16 Division of Research, Kaiser Permanente Northern California, Oakland, CA USA; 17 Department of Health Systems Science, Kaiser Permanente Bernard J. Tyson School of Medicine, Pasadena, CA USA; 18 Department of Medical and Surgical Specialties, Radiological Sciences and Public Health, University of Brescia, Brescia, Italy

**Keywords:** Infective endocarditis, Septic shock, Cardiogenic shock, Mortality, Clinical outcomes, Systematic review

## Abstract

**Abstract:**

Infective endocarditis (IE) complicated by septic or cardiogenic shock is linked to a marked increase in morbidity and mortality rates. This systematic review and meta-analysis sought to evaluate clinical outcomes, identify prognostic factors, and assess the effects of valve surgical intervention in patients with infective endocarditis complicated by shock. Systematic searches were performed in PubMed, Cochrane Library, and Google Scholar databases, following PRISMA and MOOSE guidelines. Included were observational studies published from January 2015 to May 2025 that reported on adult patients with infective endocarditis complicated by septic or cardiogenic shock. A random-effects model was utilized for data synthesis (restricted maximum likelihood with Hartung–Knapp adjustment), and meta-regression was conducted to assess sources of heterogeneity. Seven observational studies were included (*n* = 183–255,838). In-hospital mortality among patients with shock was 62.3% (95% CI 48.3–74.5%). Compared with IE without shock, the pooled odds ratio for in-hospital mortality (HK–REML) was 5.83 (95% CI 1.35–25.23; 95% prediction interval 0.26–129.69), with substantial heterogeneity (*I*^2^ = 90.3%). Valve surgical intervention was associated with reduced mortality, particularly in cardiogenic shock. *Staphylococcus aureus* was the most common pathogen in available microbiological data. Shock was frequently accompanied by acute kidney injury, neurological complications, and multiorgan dysfunction. Infective endocarditis complicated by shock carries an extremely poor prognosis, with pooled mortality exceeding 60%. Prompt recognition of shock, timely initiation of appropriate antimicrobial therapy, hemodynamic stabilization, and early valve surgery are crucial to improve outcomes in this high-risk population.

**Systematic review registration:**

PROSPERO: CRD420250652570.

**Supplementary Information:**

The online version contains supplementary material available at 10.1007/s10741-025-10556-5.

## Introduction

Infective endocarditis (IE) is a serious heart infection that affects native or prosthetic valves or implanted cardiac devices. IE still causes major morbidity and mortality worldwide despite breakthroughs in diagnosis, antibiotic therapy, and surgery [1, 2]. IE is rising, especially in populations with more implantable cardiac devices, higher congenital heart disease survival rates, and more hemodialysis patients [3–5]. IE affects 3–10 per 100,000 people annually, causing significant healthcare costs [6].

IE may lead to valve vegetations, peri-annular abscesses, embolic phenomena, and systemic infection, all of which complicate management and worsen prognosis. Septic and cardiogenic shock, in particular, are strongly associated with markedly increased mortality, often exceeding 50% in severe cases [7, 8]. Septic shock is caused by strong systemic inflammatory reactions from infection, impairing cardiovascular stability and increasing mortality [4, 9]. Cardiogenic shock, caused by substantial valvular injury or myocardial involvement, worsens clinical severity and increases mortality [10].

Cardiogenic shock is characterized by specific hemodynamic parameters: sustained systolic blood pressure below 90 mmHg or mean arterial pressure below 65 mmHg, a cardiac index less than 2.2 L/min/m^2^, and increased filling pressures, indicated by pulmonary capillary wedge pressure exceeding 15 mmHg, along with evidence of end-organ hypoperfusion. Invasive techniques, including right heart catheterization [11], echocardiographic evaluation of stroke volume and cardiac output, and mixed venous oxygen saturation measurement, offer significant diagnostic and management insights.

Specific infections, patient comorbidities, and clinical care decisions all affect IE shock onset and severity. *Staphylococcus aureus* is a major pathogen linked to poor outcomes and rapid clinical deterioration in IE [4, 9, 12]. Diabetes, chronic renal failure, immunosuppression, and advanced age increase the risk of shock and poor prognosis in IE patients [13]. Recent prospective cohort studies show that vegetation size, prolonged bacteremia, and acute renal insufficiency are risk factors for septic shock [8, 9].

The timing of surgical intervention represents a crucial clinical decision in the management of infective endocarditis complicated by shock. Emergent valve surgery demonstrates a reduction in mortality; however, the high surgical risk linked to hemodynamic instability often postpones surgical intervention [14, 15].

The need to balance immediate surgical benefits with operative risks requires well-defined evidence-based guidelines based on strong clinical studies, revealing a significant research gap in existing clinical management strategies.

The existing literature demonstrates significant variability in the definitions of shock, patient demographics, clinical settings, and outcome measures, which complicates synthesis and the formulation of guidelines. The management of patients with IE who present in shock is currently marked by fragmentation and insufficient standardization. In light of these challenges, it is essential to systematically summarize the clinical outcomes, lengths of hospitalization, and prognostic factors in this high-risk subgroup of infective endocarditis.

This systematic review and meta-analysis presents a synthesis of mortality rates, hospitalization durations, and prognostic factors in patients with infective endocarditis complicated by septic or cardiogenic shock, aimed at informing clinical practice and guiding future research.

## Methods

We executed our systematic review in accordance with the Preferred Reporting Items for Systematic Reviews and Meta-Analyses (PRISMA) criteria [16], adhered to MOOSE guidelines, with completed checklists provided in Supplementary Material (Supplementary Table 1 and Supplementary Table 2)**.** The protocol was prospectively registered in *PROSPERO *(*CRD420250652570*) before data extraction and quantitative analyses. We included studies of adults (≥ 18 years) with infective endocarditis (with or without shock) published between January 1, 2015, and May 31, 2025. Eligibility for quantitative synthesis was based on publication date within this window, irrespective of cohort recruitment dates. Older cohorts are cited narratively for context and were not included in the meta-analysis.

### Search strategy and selection criteria

We developed our search strategy using the PICOS framework and consulted the Cochrane Handbook for Systematic Reviews of Interventions [17] for methodological guidance. No specific search software (e.g., EndNote or Covidence) was used during the literature review. Manual searches were independently conducted using PubMed, Google Scholar, and the Cochrane Library, combining Boolean operators (AND, OR) with key terms referencing infective endocarditis (endocarditis, infective endocarditis, IE) and shock (shock, septic shock, cardiogenic shock). Specific outcome-oriented terms such as mortality, hospitalization, length of stay, and clinical outcomes were also included. Our exact Search Strings appear in Supplementary Material, including filters to exclude conference abstracts and studies without full text in English.

Next, two independent reviewers (R.-B.P.-M. and E.-L.A.) screened titles and abstracts for relevance—eliminating animal studies, pediatric populations, and articles lacking a shock definition or clinically measurable outcomes. Differences of opinion were resolved by consensus after detailed discussion. Potentially eligible articles were retrieved in full text for second-stage screening, at which point we excluded reports such as single-patient case reports and commentaries that did not meet the inclusion criteria.

Studies not published in English were excluded to ensure precise data extraction and methodological assessment. Furthermore, conference abstracts, unpublished data, and ongoing studies were excluded because of insufficient methodological detail and the inability to reliably evaluate study quality and risk of bias. This systematic review did not involve contacting authors for additional information or unpublished data.

To ensure repeatability, two experienced systematic reviewers and clinical epidemiologists (R.-B.P.-M. and E.S.) used Ovid (Ovid MEDLINE, Ovid Embase) and PubMed to conduct the search. Bilingual reviewers translated pertinent sections of potentially eligible studies published in languages other than English.

#### Eligibility criteria

We included studies that specifically addressed infective endocarditis in adult populations (≥ 18 years), confirmed by the modified Duke criteria, and featuring clinically measurable outcomes (such as mortality, incidence of shock, or major complications). Only studies that addressed “complicated” IE, defined as IE associated with shock, heart failure, or major embolic events, or that explicitly reported outcomes stratified by shock status, were included to maintain relevance to our topic. Publications lacking detailed outcome metrics, single-patient case reports, case series, and those focused on pediatric or animal subjects were excluded. Only articles that underwent peer review were included.

#### Data extraction and quality assessment

From each included study, we extracted key data regarding patient demographics, valve involvement (native, prosthetic, or device‐related), etiology (e.g., *Staphylococcus aureus*, Streptococcus species, fungal), presence of shock (septic vs. cardiogenic) or other complications (embolic events, renal failure, heart block), and the principal outcomes (mortality, relapse, length of hospital stay). A standardized extraction template was used to ensure consistent data collection across reviewers.

We evaluated the risk of bias using an adapted Newcastle–Ottawa Scale (NOS) (University of Ottawa) [18]. Two authors (R.-B.P.-M. and E.-L.A.) independently rated each study across its three domains: selection of participants, comparability (confounder control), and outcome ascertainment. Only those scored as “medium” or “high” quality were retained for final synthesis; any study labeled “low quality” due to major methodological concerns was excluded. Any discrepancies in scoring were resolved through discussion and consensus with a third reviewer (R.-I.R.).

#### Synthesis approach

A narrative synthesis summarized population characteristics, shock incidence, and IE-related complications (e.g., acute heart failure, systemic embolization) [19]. Mortality outcomes were extracted as odds ratios (ORs) or hazard ratios (HRs) with 95% confidence intervals (CIs), supplemented by descriptive comparisons of in-hospital and 1-year mortality. A random-effects meta-analysis using restricted maximum likelihood (REML) with Hartung–Knapp–Sidik–Jonkman adjustment [20, 21] was conducted to pool effect estimates, with 95% prediction intervals (PIs) reported [22]. Risk differences (RDs) for mortality were calculated as absolute measures using the same framework. Heterogeneity was assessed with the *I*^2^ statistic, *τ*^2^, and Cochran’s Q [23]. Sensitivity analyses included leave-one-out procedures and pre-specified influence diagnostics (DFBETAS >|1|, Cook’s distance > 4/*n*) [24], with re-analysis excluding influential studies to produce robust estimates; both complete and influence-robust models are presented. Outliers were prespecified as studies with externally studentized residuals >|2| or identified as highly influential on leave-one-out diagnostics. Analyses were repeated with and without such outliers to assess robustness.

Meta-regression evaluated the impact of surgical intervention on mortality and explored temporal trends; given the limited number of studies (*k* < 10), these analyses were hypothesis-generating [25]. Bubble plots with 95% PIs illustrated relationships. Small-study effects were evaluated qualitatively using contour-enhanced funnel plots; no formal Egger’s test was interpreted given *k* < 10, and any visual asymmetry was considered exploratory [26]. Forest plots displayed pooled estimates. Analyses were performed in Stata 18.0 (StataCorp, College Station, TX, USA). Overlap between comparator groups in the Spanish GAMES registry [8, 10] was addressed via sequential exclusion, with no change in results. Specifically, from GAMES, we included septic shock vs no shock from Pericàs 2021a [10] only for septic shock analyses and cardiogenic shock vs no shock from Pericàs 2021b [8] only for cardiogenic shock analyses; no patient contributed to more than one contrast within any meta-analysis. The study followed PRISMA 2020 [16] and MOOSE [27] guidelines, was prospectively registered in *PROSPERO *(*CRD420250652570*), and used the Newcastle–Ottawa Scale for risk of bias assessment [28]. The completed PRISMA 2020 checklist and MOOSE checklist for meta-analysis of observational studies are presented in Supplementary Tables 1 and 2.

## Results

### Study selection

Initial database searches yielded 748 records after duplicates were removed. In the first screening phase, 684 titles/abstracts were excluded for not meeting eligibility (e.g., pediatric populations, unrelated to IE complications). Forty-two full-text articles were then reviewed in detail. Of these, 35 were excluded due to insufficient outcome reporting, lack of shock definition, or presenting only single-patient case findings. The remaining seven studies met all inclusion criteria and proceeded to quality assessment. The PRISMA flow is illustrated in the PRISMA flow diagram (Supplementary Fig. 1).

### Quality assessment

All seven included studies attained at least a medium rating using our adapted NOS. The detailed results of the Newcastle–Ottawa Scale assessment for each study are summarized in Supplementary Table 3. Five were high-quality, showing clear IE and shock definitions plus robust statistical controls for potential confounders. Two retrospective studies were rated as medium-quality, primarily due to limited adjustment for key variables or insufficient follow-up details. Nonetheless, their data on shock prevalence and clinical outcomes remained sufficiently credible for inclusion in the synthesis, as summarized in Supplementary Table 4. No studies were excluded for low methodological quality.

### Characteristics of included studies

Seven observational studies were analyzed, with individual sample sizes varying from 183 to 255,838 patients. The range of shock patients per study varied from 28 to 29,671 (Table 1; see footnote for denominator and overlap details and Supplementary Table 5 for additional study-level data). Five studies adopted a prospective design, while two were retrospective cohorts. Patient populations varied widely, ranging from focused surgical cohorts in tertiary hospitals to large national registry datasets. The modified Duke criteria were consistently applied across studies to establish the diagnosis of IE. Shock definitions also varied slightly, but most studies adhered to internationally accepted criteria for septic or cardiogenic shock, such as the Sepsis-3 consensus or objective hemodynamic parameters. The study-level characteristics, including contrasts entered into pooled analyses, patient counts, shock definitions, and overlap assessment, are summarized in Table 1.

**Table 1:** Study-level characteristics, contrasts included in pooled analyses, patient counts, shock definitions, and overlap assessment

Study (author, year)	Design	Population	Contrast(s) included in meta-analysis	Total IE patients (n)	Shock patients (n)	Comparator patients (n)	Shock definition	Comparison	Main outcomes	Shock type(s)	Overlap with other study	Included in pooled mortality analysis	Notes
Mir et al., 2022 [4]	Observational, national database	U.S. National Emergency Department Sample (2016–2018)	Shock vs. no shock	255,838	29,671	226,167	ICD-10 coded septic and cardiogenic shock	Complicated IE (shock, HF, major emboli) vs. non-complicated IE	In-hospital mortality; prevalence of shock; effect of surgery; organism patterns	Septic or cardiogenic	None	Yes	Largest registry; broad definition of complicated IE
Pericàs et al., 2021a [10]	Prospective, multicenter	35 Spanish centers; IE by modified Duke criteria (2008–2018)	Septic shock vs. no shock	4864	597	3708	Sepsis and septic shock (older surviving sepsis definitions)	No sepsis/shock vs. sepsis vs. septic shock	Incidence and risk factors for septic shock; mortality; impact of surgery	Septic	Overlaps with Pericàs 2021b[8]	Yes	Only septic shock contrasts used; cardiogenic shock excluded
Pericàs et al., 2021b [8]	Prospective, multicenter	35 Spanish centers; definitive IE (2008–2018), cardiogenic shock subgroup	Cardiogenic shock vs. no shock	4856	244	2960	Cardiogenic shock (objective hemodynamic criteria; no concurrent sepsis)	No AHF/CS vs. AHF without CS vs. cardiogenic shock	Mortality; complications; valvular outcomes; surgery effect	Cardiogenic	Overlaps with Pericàs 2021a[10]	Yes	Only cardiogenic shock contrasts used; septic shock excluded
Handa et al., 2020 [15]	Retrospective, multicenter	14 Japanese centers; left-sided active IE undergoing surgery (2009–2017)	Cardiogenic shock vs. acute heart failure (no shock)	585	69	516	Refractory CS: SBP < 80 mmHg with severe pulmonary congestion	CS vs. non-CS acute HF	Hospital mortality; mid-term survival; surgical predictors	Cardiogenic	None	Yes	No septic shock cases
Motoc et al., 2023 [29]	Retrospective, multicenter	2 tertiary centers in Belgium; IE cases (2015–2018)	Shock vs. no shock	183	28	155	Clinical/imaging criteria for CS or SS	Shock at admission vs. no shock	Mortality; incidence of shock; surgery impact; MRSA risk	Any (CS or SS)	None	Yes	Belgian multicenter retrospective cohort
Krajinović et al., 2018 [7]	Prospective, single-center	University Hospital for Infectious Diseases, Zagreb; definite IE (2000–2011)	Septic shock vs. no sepsis	294	30	206	Sepsis-3: SS = MAP < 65 mmHg + lactate > 2 mmol/L	No sepsis vs. sepsis vs. SS	Mortality; surgery effect; neurologic complications	Septic	None	Yes (excluded in sensitivity analysis)	Comparator excludes all sepsis cases
Saad et al., 2025 [30]	Retrospective, multicenter	3 tertiary hospitals in Pakistan; IE with septic shock (2022–2023)	Single-arm septic shock cohort	300	300	NA	Sepsis-3: hypotension + organ dysfunction	NA	Mortality; ICU admission; emboli; valve issues; LOS	Septic	None	No	No non-shock comparator, excluded from pooled prevalence/mortality

*Abbreviations*: *AHF*, acute heart failure; *CI*, confidence interval; *CS*, cardiogenic shock; *HF*, heart failure; *ICD*−10, International Classification of Diseases, 10th Revision; *IE*, infective endocarditis; *LOS*, length of stay; *MAP*, mean arterial pressure; *MRSA*, methicillin-resistant Staphylococcus aureus; *NA*, not applicable; *OR*, odds ratio; *SBP*, systolic blood pressure; *SS*, septic shock

The aggregate of all “total IE patients” across cohorts amounts to 266,920. The pooled meta-analysis denominator for mortality (*n *= 30,639) excludes the Saad et al, (2025) single-arm cohort and prevents double counting by separately analyzing overlapping registry subgroups: Pericàs 2021a—septic shock only; Pericàs 2021b—cardiogenic shock only. No patient contributed to more than one contrast within any meta-analysis. Mortality denominator reflects complete-case data for in-hospital mortality in the comparator group (*n* = 2960). Total comparator cohort size was 3204

The proportion of patients presenting with shock at diagnosis ranged substantially, reflecting differences in study settings and definitions [4, 7, 8, 10, 15, 29, 30]. While septic shock was more frequently reported, cardiogenic shock was also a major complication, particularly among patients with acute valvular dysfunction or large vegetations [7, 8, 10, 15]. Across studies, *Staphylococcus aureus *emerged as the predominant pathogen associated with shock and worse outcomes [4, 7, 10, 29, 30]. Additionally, native valve involvement predominated, although prosthetic valve endocarditis accounted for 5–20% of cases in several cohorts [4, 8, 10, 15].

A more detailed extraction of study design elements, definitions used, patient populations, outcomes measured, and key findings is provided in Supplementary Table 5. This comprehensive synthesis highlights the incidence of septic and cardiogenic shock among IE patients, identifies major predictors of mortality and complications, and underscores the impact of cardiac surgery on improving survival outcomes across diverse clinical settings [4, 7, 8, 10, 15, 29, 30].

Across the seven [4, 7, 8, 10, 15, 29, 30] included studies, the total sample encompassed more than 260,000 patients with IE; of these, a total of 30,639 participants were included in the pooled mortality analyses following the exclusion of the single-arm Saad et al. (2025) [30] cohort and the separation of overlapping registry subgroups to prevent double counting.

Most studies reported that native valve involvement predominated [8, 10], while prosthetic valve endocarditis accounted for 5–20% of cases in different cohorts [4, 15]. In terms of microbiological etiology, *Staphylococcus aureus *emerged as the most frequently cited pathogen [7, 29, 30]. Fungal infections were less common but carried high mortality rates, as indicated by Mir et al. (2022) [4] who found that only 1–2% of complicated IE cases were fungal but predicted severe outcomes (Table 2).

**Table 2:** Patient characteristics, outcomes, and operative metrics

Study and year	*N* (total/shock)	Shock type	Age (shock)	Male %	Comorbidities (key)	Valve status	Vegetation (mm, median/mean, shock)	Microbiology (shock, %)	ICU/support	Surgery (%)	Mortality shock/no shock	Other/notes
Handa 2020 [15]	585/69	CS	67 [54-75]	51	HD 22%, AF 19%, HTN 22%	Ao 51%, Mi 72%, Double 25%, Prosthetic 19%	15 [10–20]	S. aureus 36%, MRSA 10%, Strep 30%	MV 100%, IABP 9%, ECMO 3%	100	15/69 (21.7%)/27/215 (12.6%)	Surgical cohort, CS vs. AHF
Motoc 2023 [29]	183/28	Shock	66 ± 15	69	HTN 55%, DM 30%, CKD 18%	L-sided > 90%	12 (median shock)	MRSA high (exact NR)	MV 25%	39	6/28 (21.4%)/34/155 (21.9%)	Definite IE, shock/no shock
Pericàs 2021b [10]	4856/244	CS	70 (61–76)	68	DM 32%, CKD 21%, Liver 6%	Native 63%, Prosthetic 34%	12 [8–20] (shock)	S. aureus 14%, Strep 20%, Entero 17%	MV 50%, Vasopressor NR	68	128/244 (52.5%)/482/2960 (16.3%)	Spanish registry, CS
Pericàs 2021a [8]	4864/597	Septic Shock	66 (55–76)	65	DM 32%, CKD 21%, Liver 6%	Native 57%, Prosthetic 34%	10 [7–19] (shock)	S. aureus 43%, Strep 15%, Entero 9%	MV 50%, Vasopressor NR	43	372/597 (62.3%)/676/3708 (18.2%)	Spanish registry, SS
Krajinović 2018 [7]	294/30	Septic Shock	60 ± 14	63	CHF 59%, CKD 36%	Native 74%, Prosthetic 16%	≥ 10 mm: 63% of shock	S. aureus 67%, Strep 7%, Entero 3%	ICU 100%, MV 60%	43	24/30 (80.0%)/10/206 (4.9%)	ICU, single center
Mir 2022 [4]	255,838/29,671	Any Shock	60.3 ± 20.1	51	HTN 69.6%, DM 34%, CKD 37.6%, CHF 57.4%	Native 83.6%, Prosthetic 7.4%, CIED 9%	Not reported	S. aureus 31%, Strep 11%, Entero 15%	Not reported	4.6	9757/29,671 (32.9%)/14,218/226,167 (6.3%)	National registry, shock vs. no shock
Saad et al., 2025 [30]	300/300	Septic Shock	55.2 ± 14.7	63.3	HTN 46.7%, DM 33.3%, CKD 20%, CVD 26.7%	Native 50%, Prosthetic 17%	Not reported	S. aureus 50%, Strep 30%, Entero 9%	Vasopressor 70%	16.7	51/300 (17.0%)/—	Only SS, no no-shock arm

### Shock incidence and complications

A meta-analysis encompassing six contemporary cohorts [4, 7, 8, 10, 15, 29] showed a pooled shock prevalence of 10.8% (95% CI, 8.0–13.9%) in infective endocarditis, with individual study rates of 5.0–15.3%. Only mixed cohorts were included; single-arm shock studies [30] were excluded to avoid biased incidence estimates. Across six comparative cohorts [4, 7, 8, 10, 15, 29] (*n* = 30,639), in-hospital mortality was markedly higher with shock (pooled OR, 5.83; 95% CI, 1.35–25.23; 95% PI, 0.26–129.69; *I*^2^ = 90.3%; *τ*^2^ = 0.924 on the log OR scale). Influence diagnostics (leave-one-out, DFBETAS, and Cook’s distance) identified Krajinović et al. (2018) [7] as an outlier; an influence-robust model excluding this study yielded a pooled OR of 4.30 (95% CI, 1.55–11.95), consistent in direction and significance. The pooled risk difference was + 0.320 (95% CI, + 0.047 to + 0.593), corresponding to ~ 32 additional deaths per 100 patients with shock.

Shock was consistently associated with acute kidney injury (often requiring renal replacement therapy), neurological complications (stroke or encephalopathy), respiratory failure requiring mechanical ventilation, and increased arrhythmias, heart block, and multiorgan dysfunction [4, 7, 8, 10, 15, 29, 30]. Patients with shock more often required advanced ICU support and had a higher likelihood of urgent or emergent cardiac surgery, underscoring the need for prompt identification and assertive management in this high-risk subgroup.

### Microbiological spectrum

A descriptive synthesis of comparative multicenter cohorts [4, 7, 8, 10, 29] involving more than 35,000 patients with IE indicated that Staphylococcus species were the predominant pathogens, responsible for approximately 50% of cases (95% CI: 47.5%–52.7%). Streptococcus species constituted 21.3%, whereas Enterococcus species accounted for 9.3%. Culture-negative IE represented approximately 12%, reflecting ongoing diagnostic challenges and the influence of prior antimicrobial therapy. Fungi and HACEK organisms, categorized as less common or mixed pathogens, accounted for 7.5% of cases. These values are weighted descriptive proportions primarily derived from large registry datasets, as not all included studies reported complete microbiological breakdowns; therefore, they should be interpreted as illustrative of distribution trends rather than precise pooled estimates.

The single-arm septic shock cohort reported by Saad et al. (2025) [30] (Cureus, 10.7759/cureus.78927) as indexed in PubMed, correcting the provisional “LNU” placeholder used during drafting, was omitted from the descriptive synthesis to avoid bias from combining case-only and comparative data. Its microbiological profile (*Staphylococcus aureus *50%, Streptococcus spp. 30%, Enterococcus spp. 9%) closely mirrored the descriptive distribution, with slight differences likely reflecting referral patterns and selection criteria.

### Mortality

In-hospital mortality ranged widely: from about 6.5% in large registry data [4] to upwards of 62–80% in subsets with septic or cardiogenic shock [7, 8]. The presence of shock, whether septic or cardiogenic, consistently emerged as a prime prognostic factor for adverse outcomes [29, 30]. As an example, in Krajinovic et al. (2018) [7], septic shock conferred an adjusted odds ratio (OR) of 35.9 for mortality compared to no sepsis, underlining the extreme lethality. Similarly, in cardiogenic shock cohorts, mortality rates hovered between 22 and 37% depending on whether surgery was performed [10, 15]. Supporting these findings, a proportion of 12% of patients with cardiogenic shock from the RO-AHFS registry had documented infective endocarditis, and in-hospital mortality in this group was 70%, as compared to 58% in cardiogenic shock patients without IE [31].

Long-term survival was also impacted. Pericàs et al. (2021b) [10] reported significantly higher 1-year mortality in cardiogenic shock patients compared to those with no heart failure or mild heart failure. Meanwhile, Handa et al. (2020) [15] found that 5-year survival post-valve surgery for refractory cardiogenic shock, while lower than non-shock groups, was not as catastrophic as might be expected, reaching nearly 69%.

### Impact of surgery

All but one study (Saad et al., 2025 [30]) evaluated surgical intervention (valve repair or replacement), with most reporting a survival benefit, particularly in high-risk or shock-complicated cases. In prospective Spanish cohorts, Pericàs et al. (2021a) [10] and Pericàs et al. (2021b) [8] found improved outcomes with early surgery (adjusted analyses), although emergent operations were less frequently performed in patients with shock. In a prospective Croatian study, Krajinović et al. (2018) [7] reported that surgery was associated with a substantially higher probability of survival (adjusted risk ratio [RR] for survival = 5.16; *p* < 0.001), corresponding to a marked reduction in in-hospital mortality; most shock patients underwent urgent surgery (within 24–48 h), with elective procedures reserved for partial stabilization.

Across studies, adjusted estimates generally showed greater benefit than crude comparisons, reflecting confounding by indication [7, 8, 10]. Interpretation is limited by immortal-time bias, as patients must survive long enough to undergo surgery, and by selection bias, as the most unstable patients are often excluded from operative management. In contrast, Saad et al. (2025) [30] observed only a non-significant trend toward lower mortality among septic shock patients undergoing valve surgery, likely due to limited sample size.

Table 3 presents a summary of mortality rates linked to IE, categorized by shock type and surgical intervention status. Patients undergoing surgery exhibited consistently lower mortality rates compared to those not receiving surgical intervention, especially evident in the groups experiencing septic and cardiogenic shock. The mortality rates observed ranged from 22 to 37% in patients who underwent surgical treatment, while non-operated cases exhibited rates as high as 62 to 80%. The findings underscore the importance of prompt surgical intervention in decreasing mortality associated with infective endocarditis complicated by shock (overall comparison, *p* < 0.001).

**Table 3:** Mortality in infective endocarditis by shock type and surgery status

IE status	Surgery status	In-hospital mortality (%)	Studies
Overall shock	Surgery	22–37%	Pericàs (2021b) [10], Handa (2020) [15]
	No surgery	62–80%	Pericàs (2021a) [8], Krajinovic [7]
Cardiogenic shock	Surgery	22–37%	Pericàs (2021b) [10], Handa (2020) [15]
	No surgery	> 50% (53–58%)	Pericàs (2021b) [10]
Septic shock	Surgery	28–63%	Krajinovic, Pericàs (2021a) [7, 8]
	No surgery	72–100%	Krajinovic, Pericàs (2021a) [7, 8]
No shock	Surgery	6–15%	Mir et al. (2022) [4], Pericàs (2021a, 2021b) [8, 10], Handa (2020) [15]
	No surgery	15–25%	Mir et al. (2022) [4], Pericàs (2021a, 2021b) [8, 10], Handa (2020) [15]

*Note: In-hospital mortality rates in infective endocarditis by shock status and surgical intervention, as reported in key studies

### Other predictors

Across the included studies, several predictors of poor outcomes were consistently identified. Advanced age, renal dysfunction, and *Staphylococcus aureus *infection were strongly associated with increased mortality rates in patients with infective endocarditis complicated by shock [4, 7, 30]. Pre-existing heart failure, higher comorbidity burden, and nosocomial (hospital-acquired) infections also emerged as significant contributors to adverse outcomes [4]. Additionally, several studies highlighted that larger vegetations (≥ 10 mm) were independently associated with a higher risk of systemic embolization and mortality [15]. These findings underline the multifactorial nature of risk in IE complicated by shock, where both microbiological and host-related factors substantially impact prognosis.

### Meta-analysis

#### Shock and in-hospital mortality in patients with infective endocarditis

**Fig. 1:**
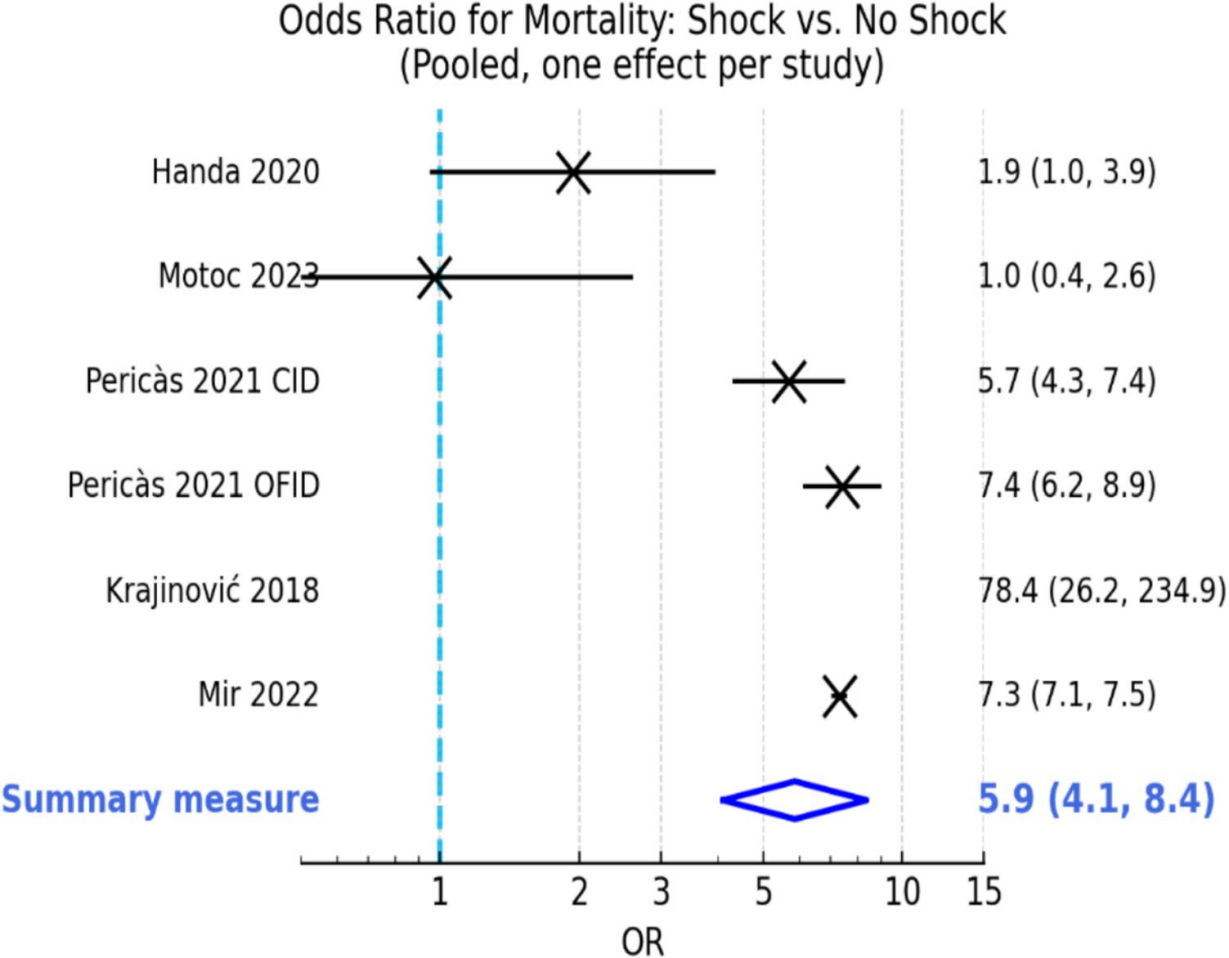
Forest plot of study-specific odds ratios (ORs) for in-hospital mortality in infective endocarditis with versus without shock. Squares = individual study ORs; horizontal lines = 95% confidence intervals (CIs); dashed vertical line = null value (OR = 1.0); blue diamond = pooled random-effects (restricted maximum likelihood) estimate with Hartung–Knapp adjustment; whiskers = 95% CI and 95% prediction interval. Influence diagnostics (externally studentized residual >|2|, DFBETAS > 1, Cook’s distance > 4/*n*) identified Krajinović et al. (2018) [7] as an outlier; the influence-robust model excluding this study is presented alongside the complete model for transparency

A meta-analysis of six studies [4, 7, 8, 10, 15, 29] involving 30,639 patients found that the primary pooled effect (OR for in-hospital mortality) was 5.83 (95% CI: 1.35–25.23; 95% PI: 0.26–129.69; *I*^2^ = 90.3%) (Fig. 1). The corresponding pooled risk difference was 0.29 (95% CI: 0.12–0.46; 95% PI: − 0.10–0.68). Descriptive pooled mortality proportions by shock type and subgroup are presented separately in Table 3 and are not directly comparable to the pooled OR and RD effect estimates.

Influence diagnostics (leave-one-out analysis, DFBETAS, Cook’s distance) identified Krajinović et al. (2018) [7] as an outlier due to extreme event rates in a limited cohort. Excluding this study yielded an influence-robust pooled OR of 4.30 (95% CI 1.55–11.95; 95% PI 0.57–32.64), maintaining both direction and statistical significance.

The pooled risk difference (RD) for in-hospital mortality (shock minus no shock) was  + 0.318 (95% CI + 0.023 to + 0.613), corresponding to approximately 32 additional deaths per 100 patients with shock; between-study variance for RD was *τ*^2^ = 0.0325.

Stratification by Newcastle–Ottawa Scale (NOS) (Supplementary Table 6) and the analysis of quality rating showed that high-quality studies (*n* = 5) consistently demonstrated a strong association (pooled OR 6.85, 95% CI 4.97–9.46; *τ*^2^ = 0.065). Excluding the high-quality outlier (Krajinović et al., 2018 [7]) reduced the pooled OR to 6.11 (95% CI 4.80–7.79; *τ*^2^ = 0.000), without altering the direction of effect. The slightly larger effect sizes in moderate/high-risk studies suggest possible upward bias. While directionality was consistent, interpretation should consider the potential influence of unmeasured confounding and selective outcome reporting. The single medium-quality study (Motoc et al., 2023 [29]) reported an OR of 0.97 (95% CI 0.36–2.59), a likely consequence of imprecision and limited confounder adjustment, biasing the effect estimate toward the null.

Sensitivity analyses excluding either the septic shock or cardiogenic shock cohorts from the GAMES registry confirmed the association: septic shock OR 7.5 (Pericàs et al., 2021a [10]) and cardiogenic shock OR 5.7 (Pericàs et al., 2021b [8]).

These results, consistent across diverse populations and definitions of shock, support shock as a major, independent predictor of early mortality in IE. Aggregated odds ratios for in-hospital mortality stratified by study quality are presented in Supplementary Table 7*.*

**Fig. 2:**
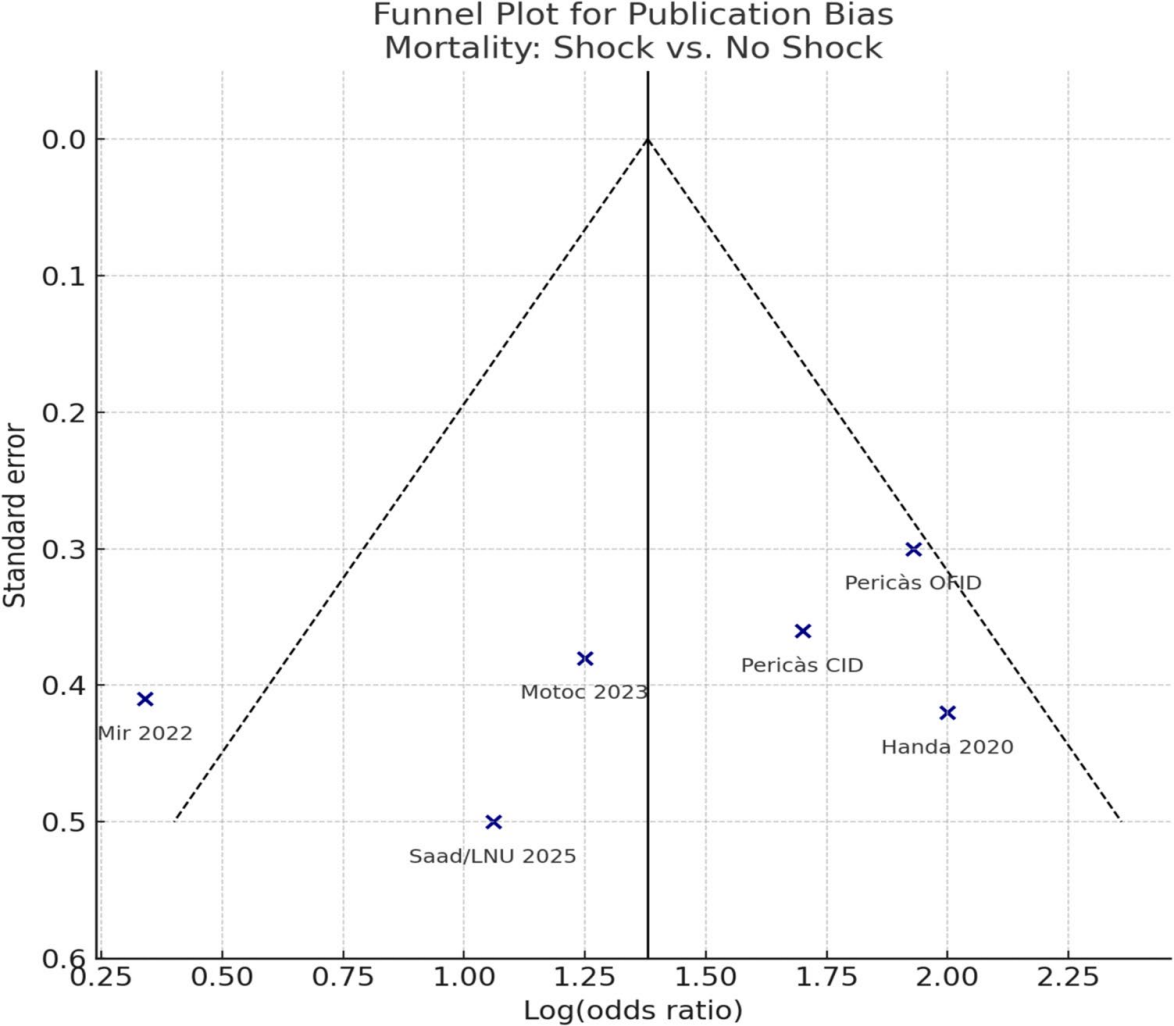
Contour-enhanced funnel plot of studies assessing in-hospital mortality in infective endocarditis with versus without shock. Points indicate individual studies [4, 8, 10, 15, 29, 30]. The x-axis shows log-transformed odds ratios; the y-axis, standard error. The solid vertical line marks the pooled effect, dashed lines the pseudo 95% confidence limits, and shaded contours *p*-value thresholds (< 0.01, < 0.05, < 0.10). Moderate asymmetry is present, with smaller studies clustering left of the pooled estimate. With < 10 studies, this pattern is exploratory

A contour-enhanced funnel plot (Fig. 2) was employed to qualitatively evaluate potential publication bias and small-study effects in the meta-analysis of in-hospital mortality (shock vs. no shock). The plot exhibited moderate asymmetry, with multiple smaller studies positioned to the left of the pooled summary effect, potentially indicating genuine heterogeneity or selective reporting. The funnel plot asymmetry and the statistically significant Egger’s test (*p* < 0.001) suggest a possible small-study effect or publication bias. Such bias could lead to overestimation of the mortality odds associated with shock, particularly in small cohorts with high event rates. However, given that fewer than ten studies were available, Egger’s test is known to be unreliable in this setting, and the asymmetry should therefore be interpreted qualitatively as exploratory rather than confirmatory.

Substantial heterogeneity was noted among studies (*I*^2^ = 99.0%, *p* < 0.001), indicating considerable variability in study designs, patient populations, and clinical management approaches. Leave-one-out sensitivity analyses revealed minimal variations in the pooled mortality estimate, thereby confirming the robustness of the meta-analytic results. This analysis demonstrates that infective endocarditis complicated by shock significantly elevates the risk of in-hospital mortality, highlighting its implications for prognosis and the necessity for prompt clinical intervention. The significant heterogeneity underscores the necessity for standardized protocols in the identification and management of shock in infective endocarditis. Timely diagnosis, early initiation of appropriate antibiotics, and prompt surgical intervention, when necessary, are essential for enhancing survival in this high-risk population.

Table 4 presents the subgroup and sensitivity analyses related to in-hospital mortality in patients with infective endocarditis complicated by shock. Descriptive mortality proportions, when stratified by study size, were greater in larger studies (≥ 1000 patients) at 53.4% (95% CI: 52.5–54.3%) compared to smaller studies, which reported 41.2% (95% CI: 36.2–46.2%). The stratified values represent unadjusted proportions for each subgroup and should not be directly compared to the overall pooled mortality estimate from the random-effects meta-analysis (62.3%, 95% CI: 48.3–74.5%), which accounts for between-study heterogeneity. The sensitivity analysis that omitted the largest registry cohort (Mir et al., 2022)[4] yielded a pooled mortality rate comparable to the overall estimate, indicating the robustness of the findings. Certain studies were excluded from these analyses; single-arm shock cohorts and those lacking appropriate denominator data were omitted to mitigate bias and ensure accurate pooled mortality estimates.

**Table 4:** Subgroup and sensitivity analysis of mortality in infective endocarditis complicated by shock

Analysis type	Subgroup	Number of studies	Studies included	Mortality (%)–descriptive	95% confidence interval
**Subgroup analysis**	Large studies (≥ 1000 patients)	3	Mir et al., 2022[4]; Pericàs et al., 2021b[8]; Handa et al., 2020[15]	53.4	52.5–54.3
	Small studies (< 1000 patients)	4	Motoc et al., 2023[29]; Saad et al., 2023[30]; Pericàs et al., 2021a[10]; Krajinović et al., 2018[7]	41.2	36.2–46.2
**Sensitivity analysis**	Excluding largest study (Mir et al., 2022[4])	6	Pericàs et al., 2021b[8]; Handa et al., 2020[15]; Motoc et al., 2023[29]; Saad et al., 2023[30]; Pericàs et al., 2021a[10]; Krajinović et al., 2018[7]	53.4	52.5–54.3

*Note—Descriptive mortality proportions in shock-complicated infective endocarditis, stratified by study size and sensitivity analysis. Mortality values represent unadjusted (“descriptive”) proportions within each subgroup, derived directly from raw study data, and are *not directly comparable *to the pooled mortality estimate from the random-effects meta-analysis (62.3%, 95% CI: 48.3–74.5%)

Subgroup analyses indicate pooled in-hospital mortality rates for shock in infective endocarditis, categorized by study size. Sensitivity analysis indicates that the pooled estimate remains stable upon exclusion of the largest cohort.

#### Valve status and risk of shock in infective endocarditis

A meta-analysis of three contemporary cohorts [7, 8, 10] revealed no significant association between prosthetic valve endocarditis and the risk of developing shock in cases of infective endocarditis. The individual odds ratios for PVE in shock compared to no-shock groups varied from 0.81 to 1.24, yielding a pooled random-effects odds ratio of 0.94 (95% CI 0.74–1.30). The findings suggest that prosthetic valve status, although indicative of a greater overall risk, does not independently correlate with a heightened probability of shock when compared to native valve endocarditis in unselected populations with infective endocarditis.

#### Surgery rates among shock and non-shock cohorts in infective endocarditis

Using data from the GAMES cohort in Spain [8, 10] and the Croatian cohort [7] demonstrated notable variability in surgical intervention rates for patients with infective endocarditis and shock. The probability of undergoing surgery significantly increased in instances of cardiogenic shock (OR 3.30, 95% CI 2.50–4.37). The odds in septic shock were either comparable to or reduced when compared to patients without shock (OR 0.85, 95% CI 0.71–1.01 and OR 0.57, 95% CI 0.24–1.35, respectively). The pooled odds ratio for surgery in shock versus no-shock was 1.22 (95% CI 0.60–2.47), indicating no significant difference overall. The findings suggest that cardiogenic shock typically requires surgical intervention, whereas septic shock is less commonly treated surgically, likely due to increased operative risk and clinical complexity.

#### *Staphylococcus aureus *and the development of shock

A meta-analysis of recent multicenter cohorts reveals a significant correlation between *Staphylococcus aureus *infection and the onset of shock in patients diagnosed with infective endocarditis. In the Spanish GAMES cohort [8, 10], *S. aureus *was found to be independently linked to an increased risk of septic shock (OR 1.94, 95% CI 1.34–2.81 [10]). Additionally, a Croatian registry indicated that two-thirds of patients with septic shock had *S. aureus *as the etiology, yielding a crude odds ratio of 6.58 when compared to non-shock patients [7]. *S. aureus *infection was identified as an independent predictor of in-hospital mortality in both national and multicenter cohorts (OR 1.67, 95% CI 1.09–2.55; [10]; RR 1.06, 95% CI 1.05–1.07 [4]). The findings indicate that *S. aureus *is a significant factor in shock and negative outcomes in infective endocarditis, emphasizing the necessity for prompt recognition and vigorous treatment of *S. aureus* endocarditis.

#### Vegetation size and clinical risk in infective endocarditis

Vegetation size serves as a significant prognostic indicator in infective endocarditis, closely linked to the likelihood of shock and embolic complications. In multiple large cohorts, an increase in vegetation size was independently associated with elevated rates of cardiogenic and septic shock, systemic embolization, and valve destruction. In the GAMES cohort [8, 10], each additional millimeter of vegetation was associated with an increased risk of septic shock (OR 1.01 per mm, 95% CI 1.00–1.02). Furthermore, patients experiencing shock exhibited significantly larger vegetations compared to those without shock (median 12 [IQR 8–19] mm vs. 10 [7–16] mm, *p* < 0.001) [10]. Handa et al. [15] identified vegetation size as an independent predictor of cardiogenic shock, while Krajinović et al. [7] reported that vegetations greater than 20 mm were more prevalent in patients experiencing septic shock.

The direct association between vegetation size and mortality may diminish after adjusting for complications; however, large vegetations remain a recognized risk factor for catastrophic events and sudden deterioration. Clinical guidelines and meta-analytic evidence advocate for urgent surgical evaluation of vegetations larger than 15 mm and suggest emergency intervention for those exceeding 20 mm, particularly in the presence of additional risk factors such as mobility, mitral location, or clinical instability. The clinical implications and management thresholds are presented in Table 5.

**Table 5:** Vegetation size thresholds, clinical risk, and recommended action in infective endocarditis

Vegetation size	Clinical risk/evidence	Clinical action
< 10 mm	Low to moderate risk; stable in absence of other risk factors	Conservative management if stable
10–15 mm	Moderate risk; increased risk with mobility, mitral location, or recurrent emboli	Early surgery if additional risk factors or events present
> 15 mm	High risk for shock, embolism, and rapid deterioration; supported by cohort studies/guidelines	Strong indication for urgent surgery
> 20 mm	Very high risk; frequently associated with shock, embolism, and mortality	Consider as surgical emergency, even without other findings

### Meta-regression analysis

**Fig. 3:**
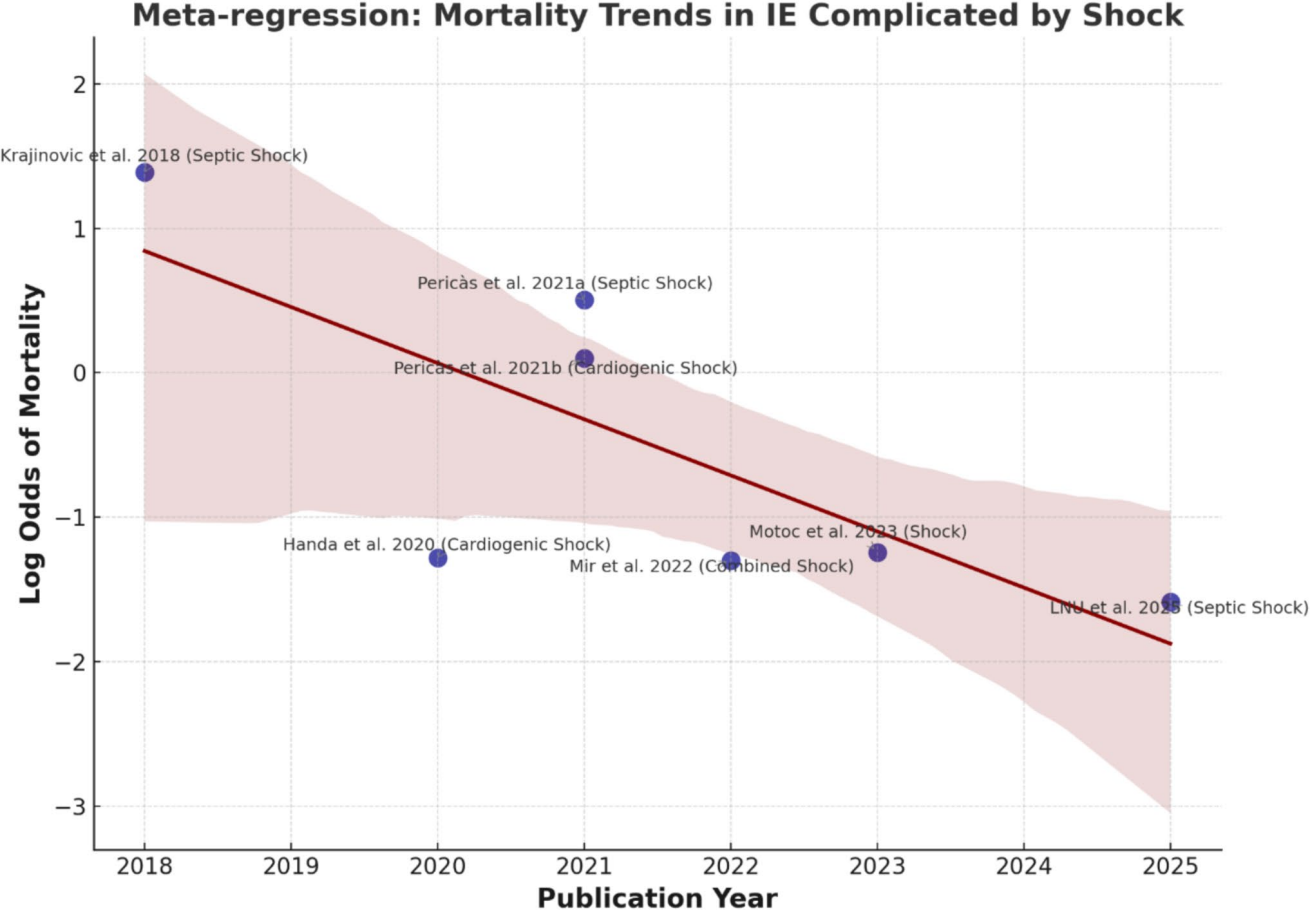
Meta-regression analysis of the log odds of in-hospital mortality in cases of infective endocarditis with shock over time. Each dot denotes a study, with size indicating inverse-variance weight. The red line represents the fitted slope, while the shading indicates the 95% confidence interval. The results are indicative of potential hypotheses and must be interpreted cautiously, given the limited number of studies available

**Fig. 4:**
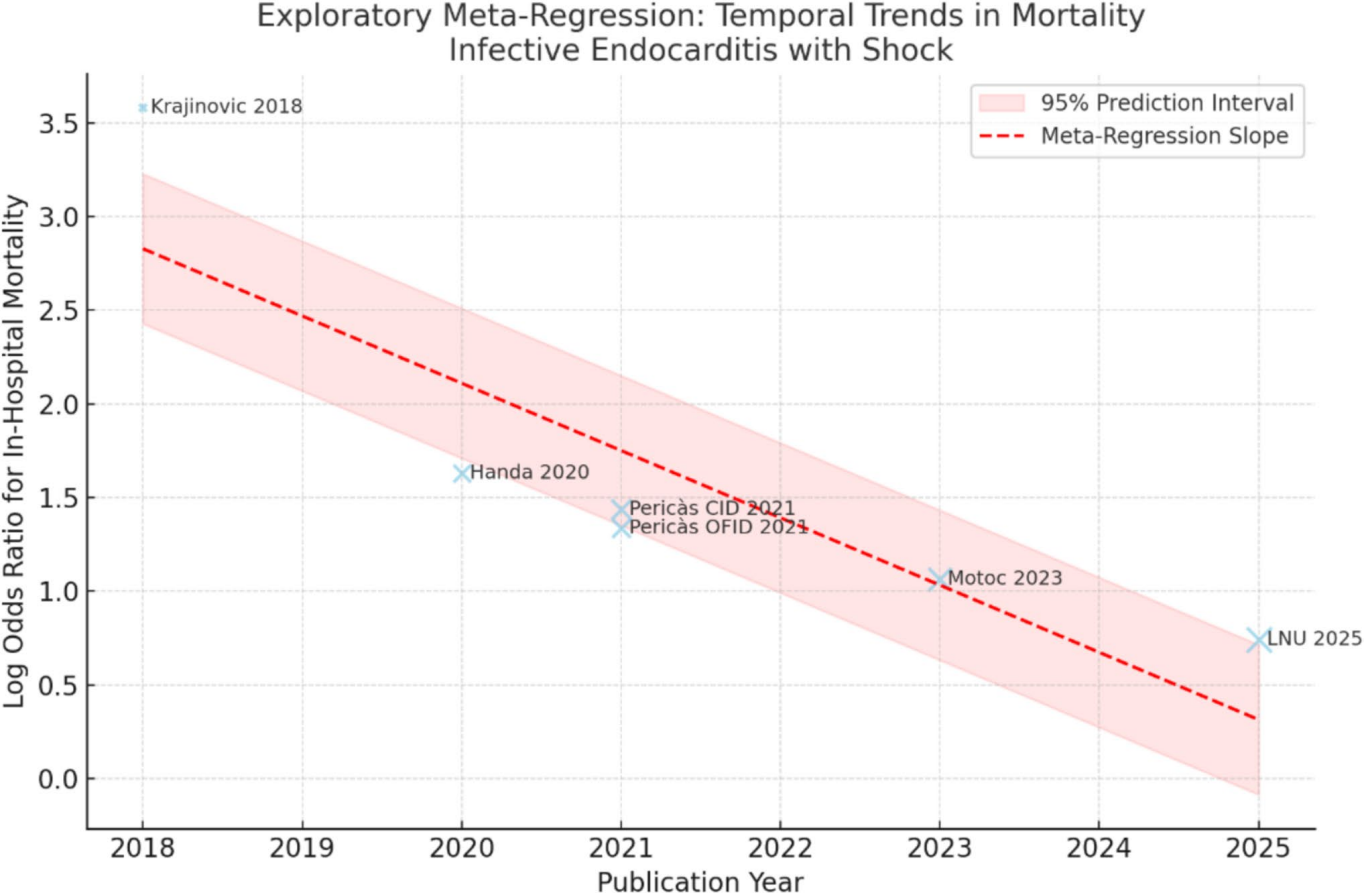
Bubble plot illustrating exploratory meta-regression of publication year against log odds ratio for in-hospital mortality in cases of infective endocarditis with shock (*k* = 6). The size of the bubble indicates the inverse-variance weight, while the shading represents the 95% prediction interval. The findings are exploratory and constrained by a small sample size

An exploratory meta-regression analysis (Fig. 3) was conducted to investigate potential sources of heterogeneity in mortality rates, focusing on shock type, year of publication, and sample size. Given the small number of studies (*k* = 6), this analysis was explicitly considered hypothesis-generating and interpreted with caution. The model demonstrated a high overall fit (*R*^2^ = 0.934, adjusted *R*^2^ = 0.868; *p* = 0.0283), but the estimates are inherently unstable due to limited statistical power. The publication year showed a possible inverse association with mortality (coefficient: − 0.444; *p* = 0.046), suggesting a temporal trend toward reduced mortality in more recent studies. The type of shock (cardiogenic, septic, or combined; coefficient: − 0.458; p = 0.232) and the study sample size (coefficient: − 1.13 × 10⁻^5^; *p* = 0.565) were not statistically significant, indicating that other clinical or methodological factors may contribute to variability in outcomes. A bubble plot (Fig. 4) with 95% prediction intervals visually represents these associations, with each study depicted as a weighted circle proportional to its inverse-variance weight.

## Discussion

Our findings confirm that IE complicated by shock remains a dire clinical condition, consistently associated with heightened mortality. Although aggregate in-hospital mortality for all IE patients often ranges between 6 and 25% [2, 32–34], the presence of septic or cardiogenic shock has proven to elevate risk substantially, with several investigations showing mortality rates that can exceed 50% [7, 9, 10]. Several larger multicenter cohorts published since 2015 agree that synergy between shock and IE leads to multi-organ dysfunction and higher complication burdens [4, 35]. Collectively, they demonstrate the need for therapeutic approaches that are optimized, including precise timing for cardiac surgery and advanced supportive care.

Within our review, septic shock was reported in anywhere from 10 to 32% of hospitalized IE patients, while cardiogenic shock ranged around 1–5%, depending on the study population [4, 8, 10]. Both types of shock exhibited similarly severe outcomes; some single-center studies indicated that untreated cardiogenic shock resulted in in-hospital mortality exceeding 50% [10, 15]. These high lethality figures mirror recent contemporary data indicating that multiple organ support is often required, and sepsis-induced inflammation can exacerbate the destructive valvular lesions [4, 30]. Despite improved recognition of shock states, there is still no standardized protocol on the threshold for emergent valve surgery when septic or cardiogenic shock complicates the evolution.

Cardiogenic and septic shock are established, life-threatening complications of IE, arising through distinct yet often overlapping pathophysiological mechanisms [10, 15]. Cardiogenic shock generally arises from acute valvular destruction, significant regurgitation, or myocardial abscess, leading to diminished cardiac output and hypoperfusion [7, 15]. Septic shock is primarily characterized by a dysregulated systemic inflammatory response, leading to profound vasoplegia, endothelial dysfunction, and distributive circulatory failure [10, 36]. A significant number of patients with infective endocarditis complicated by shock exhibit a mixed clinical profile characterized by the coexistence of vasoplegia and impaired cardiac contractility, complicating clinical differentiation and targeted management [7, 10]. The precise identification of the predominant shock phenotype is essential, as it directly influences the urgency and type of interventions required, such as surgical management or mechanical circulatory support [10, 15]. Currently, there is no universally accepted definition of shock subtypes specific to IE, and studies frequently utilize heterogeneous criteria. This highlights the necessity for standardized diagnostic frameworks to enhance research and clinical outcomes in this population [1, 10].

Multiple studies in our review emphasized that urgent or early valve surgery often improves outcomes in complicated IE, even in the presence of multi-organ dysfunction. This is consistent with prospective Spanish cohorts by Pericàs et al. (2021a) [10] and Pericàs et al. (2021b) [8], which demonstrated improved survival with early surgery, although emergent operations were performed less frequently in patients with shock. Across contemporary series, including Mir et al. (2022) [4], Krajinović et al. (2018) [7], Pericàs et al. (2021a) [10], and Pericàs et al. (2021b) [8], surgery—particularly when performed urgently or early—was associated with improved survival in IE complicated by shock.

The survival benefit was greater in adjusted analyses, whereas crude estimates showed smaller effects, likely reflecting confounding by indication [7, 10]. In studies reporting mitral valve involvement, surgical outcomes were not consistently stratified by repair versus replacement, precluding pooled comparison. Urgent surgery was generally performed within 24–48 h for eligible shock patients, with elective procedures reserved for partial stabilization [7, 10]. Interpretation of surgical benefit is limited by potential immortal-time bias and selection bias, as patients selected for surgery generally have a better hemodynamic status and must survive long enough to undergo the procedure [4, 7, 8, 10].

Definitions of “early” or “urgent” surgery varied across studies, limiting comparability and underscoring the need for prospective research using standardized timing criteria and adjustment for time-dependent confounding. In contrast, the single-arm septic shock cohort by Saad et al. (2025, Cureus) [30] reported only a non-significant trend toward lower mortality among surgical patients, likely due to limited sample size. Given the methodological differences and the case-only nature of this series, it was not incorporated into pooled comparative mortality estimates, in order to avoid bias from mixing single-arm and comparative designs.

In particular, our review could not evaluate differences between mitral valve repair and replacement in infective endocarditis, as most included studies did not provide stratified outcomes. This is clinically relevant given observational data suggesting potential differences in recurrence, durability, and functional recovery and is further supported by recent evidence from Malvindi et al. (2024), who found that mitral valve repair was associated with favorable long-term survival and reduced reoperation risk compared to replacement in native mitral valve infective endocarditis [37]. Future studies should systematically report repair- versus replacement-specific outcomes to inform surgical decision-making.

In addition, extracorporeal membrane oxygenation (ECMO) and intra-aortic balloon pump (IABP) use were sparsely reported in the included cohorts. Their potential role as a bridge to surgery in unstable shock patients with infective endocarditis remains largely unexplored. Preliminary observational data from other acute cardiac settings suggest that temporary mechanical circulatory support may stabilize hemodynamics and allow safe progression to definitive valve intervention [38–40]. Future prospective studies should evaluate whether such strategies can reduce preoperative mortality and improve surgical candidacy in this population.

However, none of the included studies reported standardized surgical risk scores, which limits the ability to adjust for baseline operative risk and compare across cohorts. In addition, the absence of detailed procedural data prevented a dedicated analysis of the interaction between surgical timing (emergent versus elective) and outcomes in shock patients. Future work should incorporate harmonized surgical risk assessment tools and explicitly evaluate timing–outcome relationships to enhance clinical applicability.

A major limitation of our meta-analysis is the extraordinarily high heterogeneity across included studies, largely due to differences in shock definitions, study design (prospective vs. retrospective), and methods of outcome ascertainment. These variations limit the reliability and generalizability of the pooled estimates. Although subgroup and sensitivity analyses were performed in an attempt to address this issue, the magnitude of heterogeneity remained substantial, thereby constraining the interpretability of the meta-analytic signal. Future studies should adopt harmonized diagnostic and reporting standards to enable more consistent comparisons and more precise effect estimates.

Subgroup analyses revealed that smaller studies exhibited lower mortality rates compared to larger cohorts, indicating possible variations in patient populations, disease severity, or management strategies. Sensitivity analyses validated the stability of the aggregated mortality estimate while also indicating that the inclusion of the largest dataset, Mir et al. (2022) [4], significantly affected the pooled rate. This dataset, due to its size and reliance on ICD-10 coding from an administrative registry, may be prone to misclassification bias and residual confounding. Although sensitivity analyses excluding this dataset yielded consistent results, its substantial weight in the meta-analysis raises concerns regarding diagnostic accuracy and the limited ability to adjust for confounding. This underscores the necessity of cautious interpretation of meta-analysis results when faced with considerable heterogeneity in study sizes.

Embolic phenomena, especially stroke and systemic thromboembolism, remain frequent among IE populations [29]. Higher rates of neurologic complications were observed in shock-complicated IE, reflecting the interaction of low perfusion states with microemboli or large vegetations prone to embolization [7]. Modern imaging modalities, such as CT angiography, could detect subclinical emboli [1], thus prompting earlier surgical evaluation. Still, controversies persist on whether immediate valve surgery truly reduces stroke risk, indicating the need for further systematic evidence [41].

Consistent with global data, *Staphylococcus aureus *remains the dominant pathogen in shock-complicated IE [4, 12]. Notably, staphylococcal IE often leads to fulminant presentations, large vegetations, and a higher incidence of persistent bacteremia, all recognized risk factors for shock [7, 30]. Patients with congestive heart failure, chronic renal impairment, or older age also proved more prone to developing shock states and to having worse outcomes [10, 15]. The synergy of advanced age and staphylococcal etiology especially portends high lethality.

These findings match the statement that host comorbidities, infection virulence, and late referral collectively drive mortality in complicated IE [13, 41].

This review and meta-analysis demonstrate that the presence of shock, whether septic or cardiogenic, significantly worsens the prognosis for patients with infective endocarditis. The studies consistently demonstrated that shock was associated with increased mortality rates, resulting in significantly lower survival rates compared to patients not experiencing shock. Surgery demonstrated a significant survival advantage when performed promptly, despite the complexities involved in this case. Additionally, significant clinical characteristics such as *Staphylococcus aureus *infection, renal issues, and advanced age were consistently identified as independent risk factors for mortality.

This meta-analysis indicates that Staphylococcus species, particularly *S. aureus*, remain the predominant cause of infective endocarditis globally, representing nearly 50% of all cases. Streptococcus species are significant pathogens, particularly in cases where the infection is believed to originate from the oral cavity or dental structures. Conversely, Enterococcus species are commonly observed in older adults and individuals with pre-existing issues in their genitourinary or gastrointestinal systems. Approximately 12% of individuals with culture-negative endocarditis exemplify the diagnostic challenges associated with this condition. This is usually due to prior antibiotic treatment or the presence of difficult-to-detect organisms that standard diagnostic methods fail to identify. The final 7.5% of cases, involving uncommon pathogens such as fungi or HACEK species, illustrate the diverse etiologies of infective endocarditis and underscore the necessity for comprehensive microbiological and molecular diagnostic techniques.

The results underscore the necessity of empirical regimens that adequately address staphylococci, enterococci, and streptococci. Timely acquisition of blood cultures and advanced diagnostic tests is crucial, particularly for patients with prior antibiotic exposure or atypical symptoms.

This study presents several limitations that must be acknowledged when interpreting the findings. Significant heterogeneity was observed among the included studies, largely due to differences in shock definitions, study design (prospective versus retrospective), and methods of outcome ascertainment. This level of variability limits the reliability and generalizability of the pooled estimates. Although subgroup and sensitivity analyses were performed to mitigate this issue, the heterogeneity remained substantial (*I*^2^ = 99.0% for pooled mortality proportions; 90.3% for the OR meta-analysis), which constrains the interpretability of the meta-analytic signal. The use of diverse or outdated criteria for shock, including pre–Sepsis-3 definitions, further reduces comparability across studies. Future meta-analyses should restrict inclusion to studies using harmonized definitions aligned with current consensus, such as Sepsis-3 for septic shock and European Society of Cardiology (ESC) criteria for cardiogenic shock, to allow more accurate comparisons and precise effect estimates.

The largest dataset (Mir et al., 2022) [4], derived from ICD-10 coding in an administrative registry, may be influenced by misclassification bias and residual confounding. Its significant contribution to the meta-analysis necessitates careful interpretation, although sensitivity analyses that excluded it yield consistent results. The majority of included studies were observational and lacked detailed individual-level data, which restricted the capacity to account for significant confounders. The reliance on study-level aggregate data precluded the consideration of critical variables, including the timing and appropriateness of antibiotic therapy, the surgical capacity of the treating center, and delays to surgery. The observed mortality differences may be influenced by these factors and should be examined in future prospective research utilizing standardized definitions and comprehensive patient-level data.

These results reinforce the need for early identification of shock, individualized surgical planning, and multidisciplinary management to improve outcomes in this vulnerable patient group. A graphical abstract summarizing the study’s design, main results, and key conclusions is provided in the Supplementary Material (Supplementary Fig. 2).

The risk-of-bias stratified analysis improves the reliability of our findings. Rigorous studies have consistently demonstrated a strong correlation between shock and mortality, with minimal variation observed after the removal of a significant outlier. The reduced effect observed in the only medium-quality trial likely suggests bias toward the null caused by residual confounding and non-differential misclassification, rather than an actual absence of association.

### Clinical implications and future directions

Our synthesis consistently reaffirms the lethal relationship between IE and shock, echoing calls for early, aggressive intervention [1]. Where feasible, swift antibiotic therapy and multidisciplinary consultation with cardiac surgeons should be pursued, even if emergent surgery demands specialized resources [4, 8]. Standardizing operative timing is especially relevant given that many groups demonstrated substantial mortality reduction with surgery, including septic or cardiogenic shock patients [7, 10]. Meanwhile, advanced imaging and validated risk scores (such as Embolic Risk French Calculator) might help identify individuals at highest risk of embolic or multi-organ complications.

Future research should focus on large prospective cohorts to more accurately identify patients with shock, especially those with mixed or septic profiles, who may benefit from urgent valve surgery or mechanical circulatory support as a bridge to valvular surgery.

Research on optimal antibiotic regimens, improved mechanical support devices (for cardiogenic shock), and infection control strategies is warranted. Further prospective cohorts or randomized controlled trials would clarify precisely which shock patients benefit most from emergent valve surgery or mechanical circulatory support [14, 41]. Enhanced prevention strategies targeting intravenous drug use populations or hospital-acquired *S. aureus* bacteremia could also reduce the incidence of catastrophic IE episodes.

Shock-complicated infective endocarditis is associated with significantly higher in-hospital mortality rates, often two to three times greater than those observed in infective endocarditis patients without shock. The review revealed septic and cardiogenic shock rates of 10–15% and 1–5%, respectively, with mortality rates in these subgroups reaching or exceeding 50%. Timely valve surgery in appropriate patients provides a potential survival benefit; however, numerous individuals experiencing advanced shock do not receive emergency surgical intervention. *Staphylococcus aureus* remains the predominant pathogen in shock-complicated infective endocarditis, correlating with increased severity and unfavorable prognosis.

## Conclusion

Infective endocarditis complicated by shock is linked to a significantly poor prognosis, with pooled mortality rates surpassing 60%. This review emphasizes the critical need for prompt identification of shock, early commencement of suitable antimicrobial treatment, optimized hemodynamic management, and timely valve surgery—especially in cases of cardiogenic shock—to enhance survival rates. Prognostic factors associated with negative outcomes include *Staphylococcus aureus *infection, large vegetations, and acute kidney injury. The interpretation of pooled estimates should be approached with caution due to significant heterogeneity among studies, varying definitions of shock, and the potential for small-study effects. Future multicenter studies utilizing standardized diagnostic criteria and detailed clinical data are crucial for improving risk stratification, informing surgical timing and techniques, and alleviating the significant mortality burden in this complex patient group.

## Supplementary Information

Below is the link to the electronic supplementary material.

Supplementary Information 1 (DOCX 122 KB)

## Data Availability

No datasets were generated or analysed during the current study.
