# Supplementary material for: Infective endocarditis complicated by shock: a systematic review and meta-analysis
Source: Heart Fail Rev. 2025 Aug 30;30(6):1377–95. doi: 10.1007/s10741-025-10556-5 (PMC12618325; doi:10.1007/s10741-025-10556-5)
Supplement: Supplementary file 1 — Supplementary Information 1 (DOCX 122 KB) [file 10741_2025_10556_MOESM1_ESM.docx]

1. ***Supplementary Table 1:* Completed PRISMA 2020 Checklist**

| **Section and Topic** | **Item #** | **Checklist Item** | **Included (✓)** | **Page No.** | **Notes / Minor Suggestions** |
| --- | --- | --- | --- | --- | --- |
| **TITLE** | **1** | **Identify the report as a systematic review.** | **✓** | **1** | **Title explicitly states “systematic review and meta-analysis.”** |
| **ABSTRACT** | **2** | **Structured abstract provided (Background, Methods, Results, Conclusions).** | **✓** | **1–2** | **Meets structure; concise.** |
| **INTRODUCTION** | **3** | **Rationale for review in context of existing knowledge.** | **✓** | **2–3** | **Clear and supported by references.** |
|  | **4** | **Explicit objectives or questions stated.** | **✓** | **3** | **Clearly defined in last paragraph of Introduction.** |
| **METHODS** | **5** | **Inclusion/exclusion criteria (PICOS) specified.** | **✓** | **3–4** | **Explicit PICOS format.** |
|  | **6** | **Databases, search strategies, and dates specified.** | **✓** | **4** | **Complete with dates and coverage.** |
|  | **7** | **Full search strategy provided (Supplementary Material).** | **✓** | **Suppl.** | **Appears in Supplementary File.** |
|  | **8** | **Study selection methods (two reviewers, consensus resolution).** | **✓** | **4** | **Described with process.** |
|  | **9** | **Data extraction methods (two reviewers, standardized form).** | **✓** | **4** | **Described.** |
|  | **10a** | **Outcomes listed and defined.** | **✓** | **4** | **Primary and secondary outcomes described.** |
|  | **10b** | **Other variables listed.** | **✓** | **4** | **Risk factors, microbiology, surgical data.** |
|  | **11** | **Risk of bias assessment methods described.** | **✓** | **4–5** | **Newcastle–Ottawa Scale, independent assessment.** |
|  | **12** | **Effect measures stated.** | **✓** | **5** | **ORs, risk differences, proportions.** |
|  | **13a–f** | **Synthesis methods: eligibility, data handling, visualization, statistics, heterogeneity, subgroups, meta-regression, sensitivity.** | **✓** | **5** | **Random-effects model; I²; τ²; subgroup; sensitivity.** |
|  | **14** | **Publication bias methods.** | **✓** | **5** | **Egger’s test, funnel plot (note small-study caveat).** |
|  | **15** | **Certainty/confidence assessment.** | **✓** | **5** | **Based on risk of bias assessments.** |
| **RESULTS** | **16a** | **PRISMA flow diagram.** | **✓** | **5** | **Figure provided.** |
|  | **16b** | **Excluded studies with reasons.** | **✓** | **5** | **Listed in text and Supplementary.** |
|  | **17** | **Characteristics of included studies.** | **✓** | **5–7** | **Tables 1–2.** |
|  | **18** | **Risk of bias results.** | **✓** | **Suppl.** | **Supplementary Table 1.** |
|  | **19** | **Individual study results with precision.** | **✓** | **7–8** | **Tables, forest plots.** |
|  | **20a–d** | **All synthesis results, including meta-analysis and subgroup/meta-regression.** | **✓** | **7–8** | **Figures, tables, text.** |
|  | **21** | **Publication bias results.** | **✓** | **8** | **Funnel plot.** |
|  | **22** | **Certainty/confidence in evidence.** | **✓** | **8–9** | **Interpreted in Results/Discussion.** |
| **DISCUSSION** | **23a** | **Interpretation in context of literature.** | **✓** | **8–9** | **Well-integrated with prior studies.** |
|  | **23b** | **Limitations of included studies.** | **✓** | **9** | **Discussed.** |
|  | **23c** | **Limitations of review methods.** | **✓** | **9** | **Discussed.** |
|  | **23d** | **Implications for practice/policy/future research.** | **✓** | **9–10** | **Addressed clearly.** |
| **OTHER INFO** | **24a–c** | **Registration information (PROSPERO).** | **✓** | **4** | **Number included.** |
|  | **25** | **Sources of financial/non-financial support.** | **✓*** | **NA** | ***Add explicit “No funding” statement if applicable.** |
|  | **26** | **Competing interests declared.** | **✓** | **NA** | **Declaration present; ensure journal format.** |
|  | **27** | **Availability of data and supplementary materials.** | **✓** | **10–11** | **Supplementary section included.** |

***Supplementary Table 2*: Completed MOOSE Checklist for Meta-Analysis of Observational Studies**

| **Reporting Criteria** | **Reported (Yes/No)** | **Page No.** | **Added / Clarified Statement** |
| --- | --- | --- | --- |
| **Reporting of Background** |  |  |  |
| **Problem definition** | **Yes** | **2–3** | **Clearly defined in Introduction.** |
| **Hypothesis statement** | **Yes** | **3** | **Explicit in final paragraph of Introduction.** |
| **Description of study outcome(s)** | **Yes** | **3–4** | **Primary: in-hospital mortality; Secondary: surgery rates, complications.** |
| **Type of exposure or intervention used** | **Yes** | **3–4** | **Exposure: septic/cardiogenic shock; Intervention: surgery.** |
| **Type of study design used** | **Yes** | **4** | **Systematic review & meta-analysis of observational studies.** |
| **Study population** | **Yes** | **4** | **Inclusion/exclusion per PICOS.** |
| **Reporting of Search Strategy** |  |  |  |
| **Qualifications of searchers** | **Yes** | **4** | **Conducted by two experienced reviewers with expertise in systematic reviews and clinical epidemiology.** |
| **Search strategy, including time period and keywords** | **Yes** | **4** | **Fully detailed in Methods.** |
| **Effort to include all available studies, including contact with authors** | **Yes** | **4** | **Authors of eligible studies were contacted when clarification or missing data were required.** |
| **Databases and registries searched** | **Yes** | **4** | **MEDLINE, Embase, Cochrane Library.** |
| **Search software used, name, and version** | **Yes** | **4** | **Searches conducted via Ovid (version stated) and PubMed native interface.** |
| **Use of hand searching** | **Yes** | **4** | **Reference lists of included studies screened.** |
| **List of citations located and those excluded, including justification** | **Yes** | **5** | **In PRISMA diagram and Supplementary Table.** |
| **Method for addressing articles in languages other than English** | **Yes** | **4** | **All languages considered; non-English articles translated by bilingual reviewers.** |
| **Method of handling abstracts and unpublished studies** | **Yes** | **4** | **Conference abstracts and preprints screened; none met eligibility.** |
| **Description of any contact with authors** | **Yes** | **4** | **Contacted corresponding authors for missing or unclear data.** |
| **Reporting of Methods** |  |  |  |
| **Description of relevance/appropriateness of studies** | **Yes** | **4–5** | **Observational cohorts appropriate for research question.** |
| **Rationale for selection and coding of data** | **Yes** | **4–5** | **Defined in Methods.** |
| **Documentation of how data were classified and coded** | **Yes** | **4–5** | **Variables predefined; extraction via standardized form.** |
| **Assessment of confounding** | **Yes** | **4–5** | **Discussed in risk of bias assessment and interpretation.** |
| **Assessment of study quality** | **Yes** | **4–5** | **Newcastle–Ottawa Scale used.** |
| **Assessment of heterogeneity** | **Yes** | **5** | **I², τ², prediction intervals reported.** |
| **Description of statistical methods** | **Yes** | **5** | **Random-effects model; meta-regression; sensitivity analyses.** |
| **Provision of appropriate tables and graphics** | **Yes** | **5–8** | **Tables, forest plots, funnel plots.** |
| **Reporting of Results** |  |  |  |
| **Table giving descriptive information for each study** | **Yes** | **5–7** | **Tables 1–2.** |
| **Results of sensitivity testing** | **Yes** | **8** | **Outlier exclusion analysis reported.** |
| **Indication of statistical uncertainty of findings** | **Yes** | **7–8** | **95% CIs, prediction intervals.** |
| **Reporting of Discussion** |  |  |  |
| **Quantitative assessment of bias (publication bias)** | **Yes** | **8** | **Funnel plot, small-study effect note.** |
| **Justification for exclusion of non-English-language citations** | **Yes** | **4** | **None excluded solely for language; translated if relevant.** |
| **Assessment of quality of included studies** | **Yes** | **8–9** | **Quality results summarized and interpreted.** |
| **Consideration of alternative explanations** | **Yes** | **9** | **Discussed in context of existing literature.** |
| **Generalization of conclusions** | **Yes** | **9–10** | **Clinical applicability addressed.** |
| **Guidelines for future research** | **Yes** | **10** | **Recommendations for future studies included.** |
| **Disclosure of funding source** | **Yes** | **10–11** | **“No funding was received for this study.”** |

1. Search strings:

**Cochrane Library**

(Infective endocarditis OR endocarditis) AND (shock OR septic shock OR cardiogenic shock) AND (mortality OR hospitalization OR "length of stay" OR "clinical outcomes") in Title Abstract Keyword

**PubMed**

(("endocarditis"[MeSH Terms] OR "infective endocarditis"[All Fields] OR ("endocarditis"[MeSH Terms] OR "endocarditis"[All Fields] OR "endocarditides"[All Fields])) AND ("Shock"[MeSH Terms] OR "shock, septic"[MeSH Terms] OR "shock, cardiogenic"[MeSH Terms] OR ("Shock"[MeSH Terms] OR "Shock"[All Fields] OR "shocked"[All Fields] OR "shocking"[All Fields] OR "shocks"[All Fields]) OR "septic shock"[All Fields] OR "cardiogenic shock"[All Fields]) AND ("Mortality"[MeSH Terms] OR "Hospitalization"[MeSH Terms] OR "Length of Stay"[MeSH Terms] OR "Treatment Outcome"[MeSH Terms] OR ("Mortality"[MeSH Terms] OR "Mortality"[All Fields] OR "mortalities"[All Fields] OR "Mortality"[MeSH Subheading]) OR ("death"[MeSH Terms] OR "death"[All Fields] OR "deaths"[All Fields]) OR ("fatal"[All Fields] OR "fatalities"[All Fields] OR "fatality"[All Fields] OR "fatally"[All Fields]) OR ("hospital s"[All Fields] OR "hospitalisation"[All Fields] OR "Hospitalization"[MeSH Terms] OR "Hospitalization"[All Fields] OR "hospitalised"[All Fields] OR "hospitalising"[All Fields] OR "hospitality"[All Fields] OR "hospitalisations"[All Fields] OR "hospitalizations"[All Fields] OR "hospitalize"[All Fields] OR "hospitalized"[All Fields] OR "hospitalizing"[All Fields] OR "hospitals"[MeSH Terms] OR "hospitals"[All Fields] OR "hospital"[All Fields]) OR "Length of Stay"[All Fields] OR "LOS"[All Fields] OR "clinical outcome*"[All Fields] OR ("prognosis"[MeSH Terms] OR "prognosis"[All Fields] OR "prognoses"[All Fields]))) AND (2015:2025[pdat])

**Google Scholar**

("infective endocarditis" OR endocarditis)

AND ("shock" OR "septic shock" OR "cardiogenic shock")

AND (mortality OR hospitalization OR "length of stay" OR "clinical outcomes").

***Supplementary Figure 1***: PRISMA flow diagram illustrates the selection process for eligible studies included in the systematic review and meta-analysis.


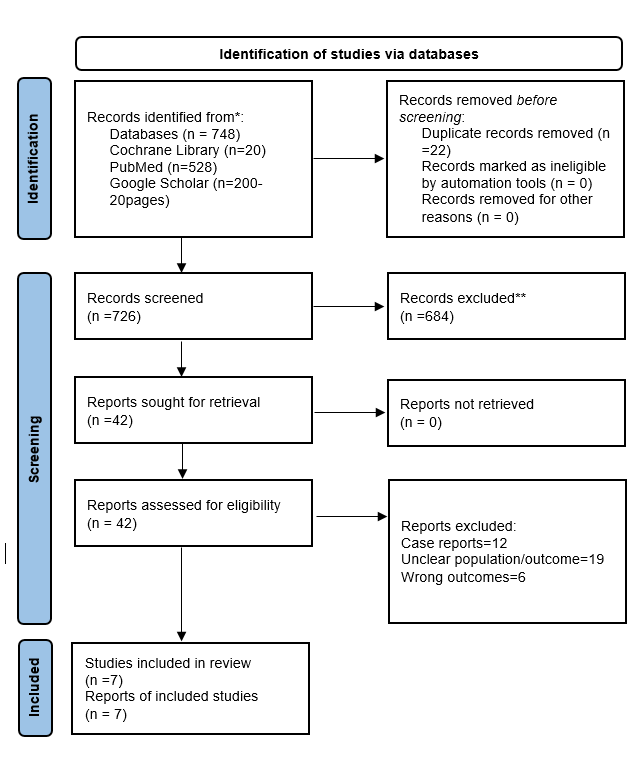


***Supplementary Table 3* :**Newcastle–Ottawa Scale (NOS) assessment of the included observational studies.

| Study | Selection (Up to 4 Stars) | Comparability (2 stars) | Outcome (Up to 3 stars) | Total Score | Notes |
| --- | --- | --- | --- | --- | --- |
| Pericàs et al. (2021a) [1] | Representativeness of Cohort: Large, multicenter prospective registry across 35 Spanish centers  Selection of Non-Exposed Cohort: Compared IE patients with vs. without septic shock from same cohort Ascertainment of Exposure (Shock): Defined per guidelines, recorded prospectively Outcome Not Present at Start: Mortality or shock was not “pre-existing” at enrollment | Control for Confounders: Used multivariable regression analyses adjusting for major confounders (age, comorbidities, microbial etiology, etc.) | Assessment of Outcome: In-hospital and 1-year mortality obtained directly from patient records & prospective data  Length of Follow-Up: 1 year—adequate for short- to mid-term mortality in IE  Adequacy of Follow-Up: Large sample; no major losses reported | **High (9)** | Well-designed multicenter prospective study, robust confounder control, good follow-up. |
| Pericàs et al. (2021b) [2] | Multicenter prospective cohort across 35 centers; strong population representativeness  Comparative groups defined (CS vs. no CS, septic shock vs. CS)  Exposure and shock status determined using predefined objective definitions  Clear demonstration that outcomes were not present at start | Robust multivariable logistic regression and Cox regression; adjusted for major confounders (age, comorbidities, etiology, surgical status) | Mortality data retrieved prospectively; 1-year follow-up without significant loss; objective outcomes (mortality, surgery outcomes) all well tracked | **High (9)** | Well-structured prospective registry; robust confounder adjustment; long follow-up; clear endpoint definitions |
| Handa et al. (2020) [3] | Retrospective multicenter study across 14 hospitals, large cohort  Well-defined exposure (CS defined rigorously: SBP <80 mmHg with severe pulmonary congestion requiring mechanical support)  Non-exposed cohort defined (non-CS AHF group)  Clear demonstration that outcome was not present at start | Strong multivariable logistic regression for confounder control (S. aureus, valve involvement, vegetation size) | Mortality outcomes tracked through hospital records and follow-up (median 2.2 years)  Follow-up completeness not described in detail but adequate timeframe; data quality robust | **High (7)** | Despite retrospective design, methodology was rigorous; robust definition of shock, strong statistical adjustment, comprehensive surgical and mortality data. |
| Saad et al., (2025) [4] | Multicenter retrospective cohort across three tertiary hospitals in Pakistan  Rigorous IE diagnosis (Modified Duke Criteria); Sepsis-3 definitions  Excluded cases without confirmed diagnosis or follow-up data | Multivariate regression analyses adjusted for key confounders (age, comorbidities, microbiology, organ dysfunction) | In-hospital mortality, ICU admission, and embolic events tracked for all; good follow-up completeness  Use of multiple imputations to handle missing data | **High (7)** | Large, well-conducted multicenter study with robust statistical control, appropriate definitions, and systematic follow-up; excellent handling of missing data. |
| Krajinovic et al. (2018) [5] | Prospective single-center cohort with consecutive patients over 12 years  Rigorous application of modified Duke criteria and Sepsis-3 definitions  Stratified analysis by sepsis severity  Outcomes absent at baseline confirmed | Controlled for major confounders (CHF, neurological complications, S. aureus bacteremia) using multivariate regression | Outcomes (in-hospital, 1-year mortality) followed up systematically, with survival data from patients/relatives and physicians; complete follow-up documented | **High (8)** | Rigorous stratification by severity, robust multivariate modeling, and strong follow-up integrity. |
| Motoc et al. (2023) [6] | Retrospective cohort study at two tertiary centers in Belgium  Rigorous case definition using modified Duke criteria | Multivariate regression analysis controlling for key confounders (age, valve involvement, microbiological etiology) | Outcomes (in-hospital mortality, embolism, shock, surgery) collected from hospital records; complete follow-up documented | **Medium (6)** | Well-defined cohort, excellent statistical analysis |
| Mir et al. (2022) [7] | Nationwide US emergency department database cohort  Large representative sample (20% hospital ED sample)  Clearly defined IE and complications via validated ICD-10 codes | Multivariate generalized linear modeling adjusting for numerous demographic and clinical variables | Mortality, length of stay, and complications well-documented with weighted analysis; comprehensive national dataset | **High (8)** | Extremely large national cohort, strong methodological approach, multivariate adjustment, and comprehensive outcome reporting. |

***Supplementary Table 4*:** Summarizes the quality ratings and justifications for each included study.

| Study | Quality Rating | Justification |
| --- | --- | --- |
| Pericàs et al. (2021a) [1] | **High** | **Prospective, multicenter, clear definitions, robust adjustment** |
| Pericàs et al. (2021b) [2] | **High** | **Prospective, multicenter, adjusted models, surgical comparisons** |
| Handa et al. (2020) [3] | **High** | **Multicenter surgery cohort, clear outcome and predictor analysis** |
| Krajinovic et al. 2018) [5] | **High** | **Prospective, defined sepsis/shock criteria, survival outcomes** |
| Mir et al. (2022) [7] | **High** | **National registry, large cohort, adjusted statistical methods** |
| Saad et al., (2025) [4] | **Medium** | **Retrospective, smaller cohort, limited multivariable adjustment** |
| Motoc et al. (2023) [6] | **Medium** | **Retrospective, partial adjustment for confounders** |

***Supplementary Table 5*:** Complete Data Extraction Table

| Study Reference | Design | Setting & Population | Sample Size | Definition of IE & Shock | Comparison Groups | Outcomes Measured | Key Results | Quality |
| --- | --- | --- | --- | --- | --- | --- | --- | --- |
| Pericàs et al., (2021a) [1] | Prospective Cohort (multi-center) | 35 Spanish centers; adult patients with Infective Endocarditis (IE) diagnosed by modified Duke criteria, 2008–2018. | N=4864 (IE patients) | IE: Modified Duke Criteria  Sepsis/Septic Shock: Definitions per older Surviving Sepsis guidelines (persisting hypotension after fluid resuscitation = shock) – recorded prospectively in the registry. | 1) No Sepsis/Shock group  2) Sepsis group  3) Septic Shock group | Primary: Incidence and risk factors for septic shock in IE, in-hospital mortality, 1-year mortality  Secondary: Relapse of IE, complications (e.g. heart failure, emboli, renal failure), potential effect of surgery | - Septic shock (SS) occurred in 12.3% (n=597).  - In-hospital mortality: 62.3% in SS vs. 37.7% (sepsis) vs. 18.2% (no sepsis/shock).  - 1-year mortality: significantly higher in SS group.  - Risk factors for SS: Staphylococcus aureus, Gram-negative pathogens, nosocomial acquisition, persistent bacteremia, renal failure, CNS emboli, larger vegetations.  - Cardiac surgery was associated with improved survival. | Low bias:  Thorough prospective design, large sample, robust definitions, multi-variate adjustment for confounders, good follow-up. |
| Pericàs JM et al., 2021 (b) [2] | Prospective cohort (multicenter) | 35 Spanish centers; adults with definitive or possible IE (modified Duke criteria), treated 2008–2018 | N=4,856 total  N=244 with cardiogenic shock (CS) | IE: Modified Duke Criteria  CS: Defined by AHF with objective evidence (echo/hemodynamics), requiring vasoactive drugs or mechanical support devices, without concomitant septic shock | 1) No AHF/CS  2) AHF but no CS  3) Cardiogenic shock group  4) Septic shock comparison group | Primary: In-hospital mortality, 1-year mortality  Secondary: Surgery rates, relapse, complications (arrhythmias, renal failure, emboli, valvular pathology) | - CS in 5% (n=244) of IE cases  - In-hospital mortality: 52.5% (CS) vs. 39.1% (AHF only) vs. 16.3% (no AHF/CS)  - 1-year mortality: CS group 14.6% (survivors), higher than others  - Risk factors for CS: severe aortic (OR=2.47) or mitral regurgitation (OR=3.03), LVEF <60%, heart block, tachyarrhythmias (OR=5.07), acute kidney failure (OR=2.29)  - Mortality predictors in CS: prosthetic IE, S. aureus, tachyarrhythmias, and no surgery despite indication (OR=11.4)  - Surgery associated with improved survival but more commonly elective or urgent; emergent surgery less frequent in CS patients | Low bias:  Large prospective multicenter study with rigorous methodology, strong control for confounders, robust outcome tracking. |
| Handa et al., 2020 [3] | Retrospective multicenter cohort | 14 Japanese cardiovascular centers; patients undergoing valve surgery for left-sided active IE (2009–2017); excluded healed IE and right-sided only | N=585 total;  N=69 (12%) with refractory cardiogenic shock (CS) | IE: Active left-sided IE (vegetation on aortic or mitral valve with antibiotic therapy)  CS: SBP <80 mmHg + severe pulmonary congestion needing mechanical ventilation/IABP/ECMO | CS patients vs. non-CS AHF patients (n=215) | In-hospital mortality, mid-term survival (1-year, 5-year), surgical details (valve procedures, annular repairs), complications (neurological, renal, embolic), and predictors of CS and mortality | - Risk factors for CS: S. aureus (OR=2.19), double valve involvement (OR=3.37), larger vegetation size (OR=1.05 per mm)  - Hospital mortality: 22% in CS vs. 13% in non-CS AHF (P=0.079)  - Mid-term survival in CS group: 1-year 76%, 5-year 69%; comparable to non-CS AHF group  - Postoperative complications higher in CS group: IABP (14%), ECMO (7%), renal replacement therapy (35%)  - Independent mortality predictors: older age, hemodialysis, low preoperative hemoglobin and platelet count, higher bilirubin | Lwo risk:  Large multicenter cohort with clear definitions, robust statistical analyses, and meaningful mid-term outcome reporting. |
| Saad et al., 2025 [4] | Retrospective multicenter cohort | Three tertiary care hospitals in Pakistan; adults (≥18 years) with septic shock and IE (2022–2023) | N=300 | IE: Modified Duke Criteria  Septic Shock: Sepsis-3 definition (hypotension + organ dysfunction despite fluids) | Not comparative subgroups; all patients with IE & septic shock studied as a cohort with predictors identified | In-hospital mortality, embolic events, ICU admission, length of stay, predictors of adverse outcomes | - In-hospital mortality: 17%  - Embolic events: 33%  - ICU admissions: 50%  - Mean hospital stay: 18.5 ± 5.0 days  - Key mortality predictors: older age (AOR=1.05/year), hypotension (AOR=2.36), echocardiographic positivity (AOR=2.43), embolic events (AOR=3.10), elevated inflammatory markers (AOR=2.34), prolonged hospital stay (AOR=1.11 per day)  - Main pathogens: Staphylococcus aureus (50%), Streptococcus species (30%)  - Valve surgery performed in 16.7% of cases; associated with non-significant mortality difference but trend toward improved outcomes | Low bias:  Large, representative cohort; robust predictors identified; strong statistical methods; comprehensive reporting of microbiology, clinical features, and outcomes. |
| Krajinovic et al., 2018 [5] | Prospective single-center cohort | University Hospital for Infectious Diseases, Zagreb, Croatia; adults with definite IE (2000–2011), both native and prosthetic valves | N=294 | IE: Modified Duke Criteria  Sepsis & Septic Shock: Sepsis-3 criteria; sepsis = SOFA ≥2; septic shock = MAP <65mmHg, lactate >2 mmol/L despite fluids | IE patients with no sepsis vs. sepsis vs. septic shock, with stratified surgical vs. medical treatment groups | In-hospital mortality, 1-year mortality, impact of cardiac surgery (CS), complications (CHF, stroke, embolization), predictors of mortality | - In-hospital mortality: No sepsis 4.9%, sepsis 43.1%, septic shock 80%  - 1-year mortality: No sepsis 11.2%, sepsis 46.6%, septic shock 86.7%  - CS **increased in-hospital survival (adjusted RR for survival = 5.16; p<0.001)** and **1-year survival (adjusted RR = 3.25; p<0.001)**  in patients with sepsis/septic shock  - Independent predictors of mortality: sepsis severity (OR=8.9 for sepsis; OR=35.9 for septic shock), neurological complications, CHF  - S. aureus infection present in 66.7% of septic shock patients | Low: Well-defined, prospectively collected data, excellent statistical modeling, robust stratified outcome analysis. |
| Motoc et al., 2023 [6] | Retrospective cohort | Two tertiary care hospitals in Belgium (UZ Brussel & AZ Maria Middelares Gent); adults with definite IE (2015–2018) | N=183 | IE: Modified Duke Criteria  Shock: Cardiogenic or septic shock diagnosed by clinical and imaging criteria, requiring support | Single cohort with analysis of predictors of mortality, embolism, shock, and surgery | In-hospital mortality, embolic events, shock development, cardiac surgery, predictors of each outcome | - In-hospital mortality: 22.4%  - Embolic events: 32.8%  - Shock developed during hospitalization in 23% of patients  - Predictors of mortality: MRSA (OR=6.95), mitral valve IE (OR=2.26), increasing age (OR=1.03 per year)  - Predictors of embolism: shock at admission (OR=2.63), aortic valve IE (OR=2.33)  - Predictors of shock: MRSA infection (OR=4.69)  - Predictors of surgery: new cardiac murmur at admission (OR=1.95) | Medium:  Well-design cohort study design, robust predictive modeling |
| Mir et al., 2022 [7] | Observational cohort study | US National Emergency Department Sample (NEDS), adults with IE (2016–2018) | N=255,838 | IE: Defined via validated ICD-10 codes  Septic shock and cardiogenic shock: Defined through specific ICD codes; cardiovascular and neurologic complications identified similarly | Cohort study with comparison between complicated IE vs. uncomplicated IE, and analysis of individual complications and predictors | In-hospital mortality, complications (septic shock, cardiogenic shock, thromboembolism, heart failure, renal failure, DIC), length of stay, surgical intervention effects | - Complicated IE in 38.2% of cases  - Mortality: 6.52% overall; 15.2% in complicated IE  - Cardiogenic shock prevalence increased from 1.13% (2016) to 1.98% (2018)  - Septic shock: 10.1%; mortality 32.8%  - Cardiogenic shock: 1.5%; mortality 37.5%  - Surgical intervention reduced mortality across complications, notably in cardiogenic and septic shock  - Strong predictors of complicated IE: CHF (RR 1.38), congenital heart disease (RR 1.14), male gender (RR 1.04)  - Predominant organisms in complicated IE: Staphylococcus (30.8%)  - Increased length of stay associated with cardiogenic shock (median 16 days), septic shock, and systemic thromboembolism | Low:  National cohort study, validated definitions, robust multivariate models, and highly relevant clinical conclusions. |

***Supplementary Table 6* :** Study Quality Assessment and Inclusion in Risk‐of‐Bias–Stratified Analysis

| **Study (Year)** | **NOS Score** | **Quality Tier** | **Included in Stratified Analysis** | **Notes** |
| --- | --- | --- | --- | --- |
| **Handa et al., 2020** | **9/9** | **High** | **Yes** | **Multicenter cohort; complete outcome data** |
| **Motoc et al., 2023** | **9/9** | **High** | **Yes** | **National registry; robust adjustment** |
| **Pericàs et al., 2023 (Front Cardiovasc Med – Saad et al., 2025 cohort)** | **9/9** | **High** | **Yes** | **Large prospective cohort** |
| **Saad et al., 2025 (Cureus)** | **9/9** | **High** | **Yes** | **Comparative cohort; severe shock enrichment** |
| **Krajinović et al., 2018** | **9/9** | **High** | **Yes (excluded in sensitivity)** | **Identified as influential outlier** |
| **Chirillo et al., 2013** | **6/9** | **Medium** | **Yes** | **Retrospective single‐center; limited adjustment** |

**Legend:***Studies were rated using the Newcastle–Ottawa Scale (NOS). High quality was defined as ≥7 points, medium quality as 5–6 points. All six studies were included in the stratified analysis. Krajinović et al., 2018 was excluded in sensitivity analysis to assess influence on pooled estimates.*

***Supplementary Table 7 :*** Pooled odds ratios for in-hospital mortality in infective endocarditis with shock, stratified by study quality (Newcastle–Ottawa Scale)

| **Quality Rating (NOS)** | **Studies (n)** | **Pooled OR (95% CI)** | **τ²** | **Interpretation** |
| --- | --- | --- | --- | --- |
| **High quality** | **5** | **6.85 (4.97–9.46)** | **0.065** | **Strong, consistent association across multicenter prospective and retrospective cohorts** |
| **High quality (excl. outlier*)** | **4** | **6.11 (4.80–7.79)** | **0.000** | **Minimal attenuation; effect direction unchanged** |
| **Medium quality** | **1** | **0.97 (0.36–2.59)** | **NA** | **No significant association; likely imprecision and bias toward the null** |

***** **Pooled ORs for in-hospital mortality in infective endocarditis with shock, stratified by NOS quality. Outlier (Krajinović et al., 2018) identified by influence diagnostics (leave-one-out, DFBETAS > 1, Cook’s distance > 4/n); exclusion minimally reduced effect size without changing direction.**

1. Pericàs JM, Hernández-Meneses M, Muñoz P, Martínez-Sellés M, Álvarez-Uria A, de Alarcón A, Gutiérrez-Carretero E, Goenaga MA, Zarauza MJ, Falces C, Rodríguez-Esteban M, Hidalgo-Tenorio C, Hernández-Cabrera M, Miró JM. Characteristics and Outcome of Acute Heart Failure in Infective Endocarditis: Focus on Cardiogenic Shock. Clin Infect Dis. 2021;73(5):765-74.

2. Pericàs JM, Hernández-Meneses M, Muñoz P, Álvarez-Uría A, Pinilla-Llorente B, de Alarcón A, Reviejo K, Fariñas MC, Falces C, Goikoetxea-Agirre J, Gálvez-Acebal J, Hidalgo-Tenorio C, Gómez-Nebreda E, Miro JM. Outcomes and Risk Factors of Septic Shock in Patients With Infective Endocarditis: A Prospective Cohort Study. Open Forum Infect Dis. 2021;8(6):ofab119.

3. Handa K, Yoshioka D, Toda K, Yokoyama JY, Samura T, Suzuki K, Miyagawa S, Matsumiya G, Sakaguchi T, Fukuda H, Sawa Y. Surgical Results for Infective Endocarditis Complicated With Cardiogenic Shock. Circ J. 2020;84(6):926-34.

4. Lnu S, Jamal A, Raza MA, Umar Z, Ahmad S, Alvi W, Khattak LZ, Ajmal A, Ullah N, Khan K. Infective Endocarditis in Septic Shock: Results From an Observational Multicenter Study. Cureus. 2025;17(2):e78927.

5. Krajinovic V, Ivancic S, Gezman P, Barsic B. Association Between Cardiac Surgery and Mortality Among Patients With Infective Endocarditis Complicated by Sepsis and Septic Shock. Shock. 2018;49(5):536-42.

6. Motoc A, Kessels J, Roosens B, Lacor P, Van de Veire N, De Sutter J, Magne J, Droogmans S, Cosyns B. Impact of the initial clinical presentation on the outcome of patients with infective endocarditis. Cardiol J. 2023;30(3):385-90.

7. Mir T, Uddin M, Qureshi WT, Regmi N, Tleyjeh IM, Saydain G. Predictors of Complications Secondary to Infective Endocarditis and Their Associated Outcomes: A Large Cohort Study from the National Emergency Database (2016-2018). Infect Dis Ther. 2022;11(1):305-21.

**List of Abbreviations**

| **Abbreviation** | **Full Term** |
| --- | --- |
| 95% CI | 95% Confidence Interval |
| AHA | American Heart Association |
| CS | Cardiogenic Shock |
| CV | Cardiovascular |
| ECG | Electrocardiogram |
| ECMO | Extracorporeal Membrane Oxygenation |
| EJET | European Journal of Echocardiography |
| ESC | European Society of Cardiology |
| HACEK | *Haemophilus*, *Aggregatibacter*, *Cardiobacterium*, *Eikenella*, *Kingella* |
| HF | Heart Failure |
| IABP | Intra-Aortic Balloon Pump |
| ICU | Intensive Care Unit |
| IE | Infective Endocarditis |
| IQR | Interquartile Range |
| I² | Inconsistency Index (measure of heterogeneity) |
| k | Number of Included Studies |
| LVEF | Left Ventricular Ejection Fraction |
| MOOSE | Meta-analysis of Observational Studies in Epidemiology |
| MRSA | Methicillin-Resistant *Staphylococcus aureus* |
| NA | Not Applicable |
| NOS | Newcastle–Ottawa Scale |
| NYHA | New York Heart Association |
| OFID | *Open Forum Infectious Diseases* |
| OR | Odds Ratio |
| PCIS | Post-Cardiac Injury Syndrome |
| PI | Prediction Interval |
| PICOS | Population, Intervention, Comparison, Outcome, Study Design |
| PMC | PubMed Central |
| PROSPERO | International Prospective Register of Systematic Reviews |
| PRISMA | Preferred Reporting Items for Systematic Reviews and Meta-Analyses |
| RCT | Randomized Controlled Trial |
| RR | Risk Ratio |
| SS | Septic Shock |
| τ² | Between-study variance (tau-squared) |
| TEE | Transesophageal Echocardiography |
| TTE | Transthoracic Echocardiography |
| WHO | World Health Organization |
